# Synthesis and Biological Evaluation of Novel Hybrid Molecules Containing Purine, Coumarin and Isoxazoline or Isoxazole Moieties

**DOI:** 10.2174/1874104501711010196

**Published:** 2017-11-30

**Authors:** Michael G. Kallitsakis, Angelo Carotti, Marco Catto, Aikaterini Peperidou, Dimitra J. Hadjipavlou-Litina, Konstantinos E. Litinas

**Affiliations:** 1 Laboratory of Organic Chemistry, Department of Chemistry, Aristotle University of Thessaloniki, Thessaloniki 54124, Greece; 2 Dipartimento di Farmacia-Scienze del Farmaco, Università degli Studi di Bari “Aldo Moro”, V. Orabona 4, I-70125 Bari, Italy; 3 Department of Pharmaceutical Chemistry, School of Pharmacy, Aristotle University of Thessaloniki, Thessaloniki 54124, Greece

**Keywords:** Modified Homo-*N*-nucleosides, Purines, Coumarins, 1,3-dipolar Cycloaddition Reaction, Antioxidant activity, Anti-lipid peroxidation activity, Alzheimer’s Disease

## Abstract

**Introduction:**

The 1,3-dipolar cycloaddition reactions of nitrile oxides formed *in situ* (in the presence of NCS and Et_3_N) from the oximes of (purin-9-yl)acetaldehyde or (coumarinyloxy)acetaldehyde with allyloxycoumarins or 9-allylpurines, respectively resulted in 3,5-disubstituted isoxazolines. The similar reactions of propargyloxycoumarins or 9-propargylpurines led to 3,5-disubstituted isoxazoles by treatment with PIDA and catalytic amount of TFA.

**Methods:**

The new compounds were tested *in vitro* as antioxidant agents and inhibitors of soybean lipoxygenase LO, AChE and MAO-B.

**Results:**

The majority of the compounds showed significant hydroxyl radical scavenging activity. Compounds **4k** and **4n** presented LO inhibitory activity.

**Conclusion:**

Compound **13**e presents an antioxidant significant profile combining anti-LO, anti-AChE and anti-MAO-B activities.

## 1 INTRODUCTION

Modified nucleosides [1, 2], coumarin derivatives [3-5], isoxazolines [6] and isoxazoles [7] represent classes of compounds with interesting broad range biological activities. Some modified nucleotides have been studied for the therapy of neurodegenerative disorders [8]. Coumarin derivatives have also been tested as acetylcholinesterase/ monoamine oxidase inhibitors for the treatment of Alzheimer’s Desease [9, 10].

In continuation to our recent studies on hybrid molecules with purine and coumarin moieties [11-13], on coumarin derivatives [3, 14, 15] and on modified nucleosides [16, 17], we present here the synthesis of new conjugated molecules as modified nucleosides, combining the coumarin and purine moieties through isoxazolines or isoxazoles as spacers. The new compounds were investigated for their antioxidant profile [free radical scavengers, lipid peroxidation and lipoxygenase (LO) inhibitors] as well as for their activity to ChEs and MAO enzymes searching for multipotent compounds.

## 2 MATERIALS AND METHODS

### 2.1 Chemistry

Some characteristic syntheses and selected data are given below:

#### 2.1.1 General procedure. 1,3-Dipolar cycloaddition reactions of (purin-9-yl)acetaldehyde oximes with alkenyloxycoumarins. Synthesis of 4-methyl-6-({3-[(6-piperidin-1-yl-9H-purin-9-yl)methyl]-4,5-dihydroisoxazol-5-yl}methoxy)-2H-chromen-2-one (4a)

In the solution of oxime **2a** (41 mg, 0.16 mmol) in dry DMF (5 ml) NCS (32 mg, 0.22 mmol) was added under stirring in portions during 1 h. The resulted mixture was stirred for 30 min. The allyloxycoumarin **3a** (32 mg, 0.16 mmol) and Et_3_N (0.03 ml, 16 mg, 0.16 mmol) were then added and the mixture was stirred at r.t. for 24 h under N_2_ atmosphere. The mixture was filtered, the solid was washed with DCM and the filtrate was evaporated. The residue was chromatographed in a column [hexane/ethyl acetate (2:1)] and gave after the elution of starting coumarin **3a** (5 mg, 16%) the isoxazoline **4a**, 51 mg, (68% yield). White crystals, m.p.180-182°C (ethyl acetate); IR (Nujol): 3020, 1715, 1620, 1570 cm^-1^; ^1^H-NMR (CDCl_3_, 500 MHz) δ 1.63-1.80 (m, 6H), 2.40 (d, 3H, *J*=1.2 Hz), 2.98 (dd, 1H, *J_1_*=7.2 Hz, *J_2_*=17.1 Hz), 3.09 (dd, 1H, *J_1_*=11.2 Hz, *J_2_*=17.1 Hz), 4.05 (d, 2H, *J*=4.3 Hz), 4.17-4.29 (m, 4H), 4.93-5.05 (m, 1H), 5.15 (s, 2H), 6.29 (d, 1H, *J*=1.2 Hz), 6.99-7.05 (m, 2H), 7.23 (d, 1H, *J*=8.8 Hz), 7.80 (s, 1H), 8.32 (s, 1H); ^13^C-NMR (CDCl_3_, 125 MHz) δ 18.6, 24.7, 26.1, 37.5, 39.8, 45.6, 69.7, 78.6, 109.3, 115.7, 118.1, 119.2, 119.6, 120.6, 137.6, 150.6, 151.7, 152.6, 153.9, 154.3, 154.8, 160.7, 161.2; MS (ESI): *m/z* 475 [M+H]^+^, 497 ;[M+Na]^+^; Anal. Calcd (%) for C_25_H_26_N_6_O_4_: C, 63.28; H, 5.52; N, 17.71. Found: C, 63.17; H, 5.47; N, 17.86.

#### 2.1.2 General Procedure. 1,3-Dipolar Cycloaddition Reactions of (Purin-9-yl)Acetaldehyde Oximes with Coumarinyl Acrylates. Synthesis of 4-Methyl-2-oxo-2H-chromen-6-yl 3-[(6-piperidin-1-yl-9H-purin-9-yl)methyl]-4,5-Dihydroisoxazole-5-Carboxylate (4k)

A solution of oxime **2a** (62 mg, 0.24 mmol) in methanol (2 ml) was added dropwise during 1.5 h at r.t. in a mixture of acrylate **3e** (60 mg, 0.26 mmol), PIDA (84 mg, 0.26 mmol) and TFA (4 μl, 5.7 mg, 0.05 mmol) in methanol (3 ml). The mixture was stirred for 4 h at r.t.. Then, the solvent was evaporated and the residue was separated by column chromatography [hexane/ethyl acetate (2:1)] to give the aldehyde **1a** (10 mg, 17%) followed by the isoxazoline **4k**, 83 mg (71% yield). White crystals, m.p.179-181°C (ethyl acetate); IR (KBr): 3027, 2912, 2834, 1719, 1702, 1621, 1589 cm^-1^; ^1^H-NMR (CDCl_3_, 500 MHz) δ 1.69-1.77 (m, 6H), 2.41 (s, 3H), 3.38-3.47 (m, 2H), 4.22-4.34 (m, 4H), 5.25 (s, 2H), 5.29 (dd, 1H, *J_1_*=6.9 Hz, *J_2_*=10.7 Hz), 6.32 (s, 1H), 7.21-7.28 (m, 2H), 7.36 (d, 1H, *J*=8.7 Hz), 7.81 (s, 1H), 8.35 (s, 1H); ^13^C-NMR (CDCl_3_, 125 MHz) δ 18.7, 24.5, 26.1, 39.4, 39.8, 46.6, 78.2, 116.0, 117.0, 118.2, 119.3, 120.7, 124.7, 137.8, 146.0, 149.1, 150.7, 151.3, 151.5, 153.3, 154.1, 160.1, 167.9; MS (ESI): *m/z* 489 [M+H]^+^; Anal. Calcd (%) for C_25_H_24_N_6_O_5_: C, 61.47; H, 4.95; N, 17.20. Found: C, 61.58; H, 4.92; N, 17.12.

#### 2.1.3 General procedure. 1,3-Dipolar cycloaddition reactions of (coumarinyl)acetaldehyde oximes with 9-allylpurines. Synthesis of 4-methyl-6-({5-[(6-piperidin-1-yl-9H-purin-9-yl)methyl]-4,5-dihydroisoxazol-3-yl}methoxy)-2H-chromen-2-one (11a)

In the solution of oxime **9a** (37 mg, 0.16 mmol) in dry DMF (5 ml) NCS (32 mg, 0.22 mmol) was added under stirring in portions during 1 h. The resulted mixture was stirred for 30 min. The allylpurine **10a** (39 mg, 0.16 mmol) and Et_3_N (0.03 ml, 16 mg, 0.16 mmol) were then added and the mixture was stirred at r.t. for 24 h under N_2_ atmosphere. The mixture was filtered, the solid was washed with DCM and the filtrate was evaporated. The residue was chromatographed in a column [hexane/ethyl acetate (2:1)] and gave after the elution of starting purine **10a** (5 mg, 13%) the isoxazoline **11a**, 53 mg, (70% yield). White crystals, m.p.148-150°C (ethyl acetate); IR (Nujol): 3080, 1720, 1630, 1565 cm^-1^; ^1^H-NMR (CDCl_3_, 500 MHz) δ 1.69-1.79 (m, 6H), 2.40 (s, 3H), 3.07 (dd, 1H, *J_1_*=6.6 Hz, *J_2_*=17.9 Hz), 3.24 (dd, 1H, *J_1_*=10.9 Hz, *J_2_*=17.9 Hz), 4.23-4.33 (m, 4H), 4.39 (dd, 1H, *J_1_*=5.2 Hz, *J_2_*=14.0 Hz), 4.46 (dd, 1H, *J_1_*=5.4 Hz, *J_2_*=14.0 Hz), 4.76 (s, 2H), 5.03-5.14 (m, 1H), 6.31 (s, 1H), 6.97-7.03 (m, 2H), 7.25 (d, 1H, *J*=8.5 Hz), 7.88 (s, 1H), 8.37 (s, 1H); ^13^C-NMR (CDCl_3_, 125 MHz) δ 18.7, 24.0, 26.3, 38.3, 45.6, 49.3, 63.2, 78.6, 109.4, 114.9, 116.0, 118.3, 119.0, 120.8, 124.4, 138.9, 145.6, 149.6, 151.8, 152.3, 154.0, 160.3, 162.1; MS (ESI): *m/z* 475 [M+H]^+^, 497 [M+Na]^+^; Anal. Calcd (%) for C_25_H_26_N_6_O_4_: C, 63.28; H, 5.52; N, 17.71. Found: C, 63.35; H, 5.47; N, 17.59.

#### 2.1.4 General procedure. 1,3-Dipolar cycloaddition reactions of (purin-9-yl)acetaldehyde oximes with propargyloxycoumarins. Synthesis of 4-methyl-6-({3-[(6-piperidin-1-yl-9H-purin-9-yl)methyl]isoxazol-5-yl}methoxy)-2H-chromen-2-one (13a)

TFA (4 μl, 5.7 mg, 0.05 mmol) was added to the solution of propargyloxycoumarin **12a** (56 mg, 0.26 mmol) and PIDA (84 mg, 0.26 mmol) in methanol (3 ml). Then, in the resulted mixture, a solution of oxime **2a** (62 mg, 0.24 mmol) in methanol (2 ml) was transferred dropwise during 1.5 h and the mixture was stirred at r.t. for 4 h. The solvent was evaporated and the solid residue was separated by column chromatography [hexane/ethyl acetate (2:1)] followed by PTLC (ethyl acetate) to give the aldehyde **1a** (5 mg, 9%) and the isoxazole **13a** (73 mg, 64%). White crystals, m.p.151-152°C (DCM); IR (Nujol): 3030, 1710, 1620, 1570 cm^-1^; ^1^H-NMR (CDCl_3_, 500 MHz) δ 1.68-1.82 (m, 6H), 2.40 (s, 3H), 4.21-4.38 (m, 4H), 5.16 (s, 2H), 5.48 (s, 2H), 6.32 (s, 1H), 6.44 (s, 1H), 7.06-7.17 (m, 2H), 7.29 (d, 1H, *J*=8.7 Hz), 7.83 (s, 1H), 8.40 (s, 1H); ^13^C-NMR (CDCl_3_, 125 MHz) δ 18.6, 26.2, 29.7, 38.9, 47.2, 62.1, 103.4, 109.8, 116.0, 118.3, 119.3, 119.6, 120.8, 138.1, 148.9, 150.1, 151.5, 152.2, 154.0, 159.6, 160.5, 160.7, 168.8; MS (ESI): *m/z* 473 [M+H]^+^, 495 [M+Na]^+^; Anal. Calcd (%) for C_25_H_24_N_6_O_4_: C, 63.55; H, 5.12; N, 17.79. Found: C, 63.62; H, 5.17; N, 17.63.

#### 2.1.5 General procedure. 1,3-Dipolar cycloaddition reactions of [(2-oxo-2H-chromen-7-yl)oxy]acetaldehyde oxime (9d) with propargylpurines. Synthesis of 7-({5-[(6-piperidin-1-yl-9H-purin-9-yl)methyl]isoxazol-3-yl}methoxy)-2H-chromen-2-one (15a)

TFA (4 μl, 5.7 mg, 0.05 mmol) was added to the solution of propargylpurine **14a** (63 mg, 0.26 mmol) and PIDA (84 mg, 0.26 mmol) in methanol (3 ml). Then, in the resulted mixture, a solution of oxime **9d** (53 mg, 0.24 mmol) in methanol (2 ml) was transferred dropwise during 1.5 h and the mixture was stirred at r.t. for 4 h. The solvent was evaporated and the solid residue was separated by column chromatography [hexane/ethyl acetate (2:1)] followed by PTLC (ethyl acetate) to give the aldehyde **8d** (5 mg, 11%) and the isoxazole **15a** (62 mg, 56%). White crystals, m.p. 140-142°C (DCM); IR (Nujol): 3040, 1725, 1595 cm^-1^; ^1^H-NMR (CDCl_3_, 500 MHz) δ 1.65-1.77 (m, 6H), 4.18-4.35 (m, 4H), 5.16 (s, 2H), 5.52 (s, 2H), 6.25 (d, 1H, *J*=9.5 Hz), 6.41 (s, 1H), 6.80-6.93 (m, 2H), 7.36 (d, 1H, *J*=9.2 Hz), 7.60 (d, 1H, *J*=9.5 Hz), 7.85 (s, 1H), 8.37 (s, 1H); ^13^C-NMR (CDCl_3_, 125 MHz) δ 24.7, 26.2, 38.7, 47.0, 62.0, 102.3, 103.1, 112.7, 113.5, 114.0, 119.6, 129.1, 137.8, 143.1, 149.7, 151.7, 153.4, 155.9, 160.4, 160.7, 161.0, 166.8; MS (ESI): *m/z* 459 [M+H]^+^, 497 [M+K]^+^; Anal. Calcd (%) for C_24_H_22_N_6_O_4_: C, 62.87; H, 4.84; N, 18.33. Found: C, 62.93; H, 4.78; N, 18.17.

#### 2.1.6 General procedure. 1,3-Dipolar cycloaddition reactions of [(2-oxo-2H-chromen-7-yl)oxy]acetaldehyde oxime (9d) with vinylpurines. Synthesis of 7-{[5-(6-piperidin-1-yl-9H-purin-9-yl)-4,5-dihydroisoxazol-3-yl]methoxy}-2H-chromen-2-one (18a)

TFA (4 μl, 5.7 mg, 0.05 mmol) was added to the solution of vinylpurine **17a** (60 mg, 0.26 mmol) and PIDA (84 mg, 0.26 mmol) in methanol (3 ml). Then, in the resulted mixture, a solution of oxime **9d** (53 mg, 0.24 mmol) in methanol (2 ml) was transferred dropwise during 1.5 h and the mixture was stirred at r.t. for 4 h. The solvent was evaporated and the solid residue was separated by column chromatography [hexane/ethyl acetate (2:1)] followed by PTLC (ethyl acetate) to give the aldehyde **8d** (7 mg, 15%) and the isoxazoline **18a** (71 mg, 66%). White crystals, m.p. 197-199°C (ethyl acetate); IR (Nujol): 3040, 1710, 1585 cm^-1^; ^1^H-NMR (CDCl_3_, 500 MHz) δ 1.67-1.81 (m, 6H), 3.68-3.75 (m, 2H), 4.22-4.34 (m, 4H), 5.06 (d, 1H, *J*=12.8 Hz), 5.12 (d, 1H, *J*=12.8 Hz), 6.29 (d, 1H, *J*=9.6 Hz), 6.78-6.85 (m, 1H), 6.89-6.95 (m, 2H), 7.41 (d, 1H, *J*=9.1 Hz), 7.63 (d, 1H, *J*=9.6 Hz), 7.77 (s, 1H), 8.29 (s, 1H); ^13^C-NMR (CDCl_3_, 125 MHz) δ 24.7, 26.2, 41.7, 46.9, 63.1, 84.4, 102.5, 112.4, 113.7, 114.3, 120.3, 129.2, 136.0, 143.0, 149.7, 152.0, 153.4, 155.8, 155.9, 160.6, 160.7; MS (ESI): *m/z* 447 [M+H]^+^; Anal. Calcd (%) for C_23_H_22_N_6_O_4_: C, 61.87; H, 4.97; N, 18.82. Found: C, 61.95; H, 4.93; N, 18.72.

### 2.2 Biology

#### 2.2.1 Materials and Methods

All the reagents used were commercially available by Merck, 1,1-diphenyl-2-picrylhydrazyl (DPPH), nordihydroguairetic acid (NDGA) were purchased from the Aldrich Chemical Co. Milwaukee, WI, (USA). Soybean Lipoxygenase, linoleic acid sodium salt, were obtained from Sigma Chemical, Co. (St. Louis, MO, USA). Trolox were purchased by Fluka A.G. For *in vitro* determination a UV-Vis Shimadzu Spectrophotometer was used.

#### 2.2.2 In vitro

In the *in vitro* assays each experiment was performed at least in triplicate and the standard deviation of absorbance was less than 10% of the mean.

##### 2.2.2.1 Determination of the reducing activity of the stable radical 1, 1-diphenyl-picrylhydrazyl (DPPH) [14]

To a solution of DPPH (100μM) in absolute ethanol an equal volume of the compounds dissolved in ethanol was added. As control solution ethanol was used. The concentration of the solutions of the compounds was 100μM. After 20 and 60 min at room temperature the absorbance was recorded at 517 nm (Table **5**). NDGA was used as a standard.

##### 2.2.2.2 Competition of the tested compounds with DMSO for hydroxyl radicals [37]

The hydroxyl radicals generated by the Fe ^3+^ /ascorbic acid system, were detected according to Nash, by the determination of formaldehyde produced from the oxidation of DMSO. The reaction mixture contained EDTA (0.1 mM), Fe ^3+^ (167 μM), DMSO (33 mM) in phosphate buffer (50 mM, pH 7.4), the tested compounds (concentration 0.1mM) and ascorbic acid (10 mM). After 30 min of incubation (37°C) the reaction was stopped with CCl_3_COOH (17% w/v) (Table **5**). Trolox was used as a standard.

##### 2.2.2.3 Inhibition of linoleic acid lipid peroxidation [14]

Production of conjugated diene hydroperoxide by oxidation of linoleic acid sodium salt in an aqueous solution was monitored at 234 nm. 2,2’-Azobis(2-amidinopropane) dihydrochloride (AAPH) was used as a free radical initiator. 10 μl of the 16 mM linoleic acid sodium salt solution was added to the UV cuvette containing 0.93 ml of 0.05 M phosphate buffer, pH 7.4 prethermostated at 37°C. The oxidation reaction was initiated at 37°C under air by the addition of 50 μl of 40 mM AAPH solution. Oxidation was carried out in the presence of 10 μl of the examined compounds (stock solution in DMSO). In the assay without antioxidant, lipid oxidation was measured in the presence of the same level of DMSO. The rate of oxidation at 37°C was monitored by recording the increase in absorption at 234 nm caused by conjugated diene hydroperoxides (Table **5**). Trolox was used as a standard.

##### 2.2.2.4 Soybean lipoxygenase inhibition study *in vitro* [14]

*In vitro* study was evaluated as reported previously. The tested compounds dissolved in ethanol were incubated at room temperature with sodium linoleate (0.1 mM) and 0.2 ml of enzyme solution (1/9 x10^-4^ w/v in saline). The conversion of sodium linoleate to 13-hydroperoxylinoleic acid at 234 nm was recorded and compared with the appropriate standard inhibitor nordihydroguaretic acid (IC_50_ 5.5 μM). Several concentrations were used for the determination of IC_50_ values (Table **5**).

##### 2.2.2.5 Inhibition Study on ChEs *in vitro*

*In vitro* inhibition of electric eel acetylcholinesterase (eeAChE; 463 U/mg, Sigma) and equine serum butyrylcholinesterase (esBChE; 13 U/mg, Sigma) was investigated with a 96-well plate procedure based on the classical Ellman’s spectrophotometric test, as already described [35].

##### 2.2.2.6 Inhibition Study on MAOs *in vitro*

Inhibition of rat monoamine oxidase A and B was studied by means of a spectrofluorimetric method as previously detailed [38].

## 3 RESULTS AND DISCUSSION

### 3.1 Chemistry

**Scheme 1:**
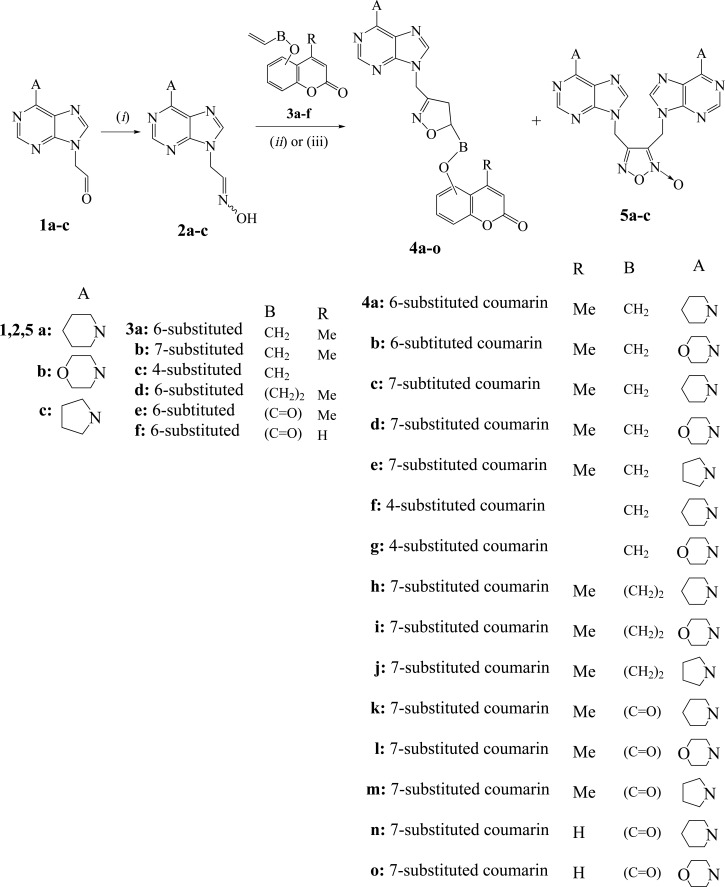
Reagents and conditions: *(i)* NH_2_OH.HCl (1 equiv.), CH_3_COONa (anh. 0.41 equiv.), H_2_O, EtOH, 80^0^C, 2.5 h; *(ii)* DMF (dry), NCS (1.4 equiv.) in portions during 1 h, N_2_, r.t. 30 min, **3** (1 equiv.), Et_3_N (1 equiv.), r.t. 24 h, for **4a-k**; *(iii)* TFA (0.2 equiv.), **3** (1.1 equiv.), PIDA (1.1 equiv.) in MeOH, **2** (1 equiv.) in MeOH (dropwise during 1 h), r.t. 4 h, for **4k-o**.

The reactions studied and the title new compounds received are depicted in Schemes (**1**-**5**). The nitrile oxide, generated from the oxime **2a** [18] by chlorination with NCS in DMF solution followed by addition of Et_3_N in an one-pot procedure, was treated with the allyloxycoumarin **3a** [19] under stirring for 24 h and Ar atmosphere (Scheme **1**) to give the isoxazoline **4a** in 68% yield (Table **1**, entry 1). The isoxazoline **4a** has the expected regiochemistry [18] as indicated by HMBC experiments. There is correlation between the protons of NC*H*_2_ group [5.15 ppm (s, 2H)] with the carbon of 4-*C*H_2_ (isoxazoline) (37.5 ppm in ^13^C-NMR) and not with the 5-*C*H (isoxazoline) (78.6 ppm).

The analogous reaction of the 6-allyloxycoumarin **3a** with the nitrile oxide formed *in situ* from the oxime **2b** (prepared by treatment of aldehyde **1b** [13] in ethanol-water with NH_2_OH.HCl in the presence of anhydrous CH_3_COONa at 80°C for 2.5 h) resulted in the isoxazoline **4b** in 68% yield (Table **1**, entry 2). The 1,3-dipolar cycloaddition reactions of 7-allyloxycoumarin **3b** [37, 38] with nitrile oxides resulted from the oximes **2a,b** and **2c** (synthesized from the aldehyde **1c** [13]) gave the isoxazolines **4c,d,e** respectively (Table **1**, entries 3-5). The one-pot reactions of 4-allyloxycoumarin (**3c**) [12, 20] with the nitrile oxides of oximes **2a,b** led to the isoxazolines **4f,g** in 73% and 70% yield respectively (Table **1**, entries 6,7). The isoxazolines **4h-j** isolated from the reactions of 7-butenyloxycoumarin **3d** [12] with the nitrile oxides formed from oximes **2a-c** respectively (Table **1**, entries 8-10). The isoxazoline **4h** has the same regiochemistry, like the others, as the protons of NC*H*_2_ group [5.16 ppm (s, 2H)] in HMBC experiments are correlated with the carbon of 4-*C*H_2_ (isoxazoline) (38.7 ppm in ^13^C-NMR) and not with the 5-*C*H (isoxazoline) (78.9 ppm). In all the above experiments none of the possible furoxans **5a-c** was detected.

In the case of (coumarin-6-yl)acrylate **3e** [12] the one-pot procedure with NCS, Et_3_N and oxime **2a** led to the isoxazoline **4k** in only 34% yield along with the furoxan **5a** (27%) [18]. When the reaction of acrylate **3e** with the oxime **2a** was performed with PIDA as oxidizing agent in the presence of catalytic amount of TFA in methanol under stirring for 4 h, the yield for the isoxazoline **4k** increased to 71% (Table **1** entry 11). No furoxan **5a** was detected. The reactions of acrylates **3e,f** with the oximes **2b,c** and **2a,b** respectively in the presence of PIDA and TFA gave the isoxazolines **4l-o** (Table **1**, entries 12-15). No furoxans **5b,c** were detected by this method.

Another way is presented in Scheme (**2**) for the synthesis of the hybrid compounds **11a-g** using the 1,3-dipolar cycloaddition reactions of nitrile oxides, formed *in situ* from the (coumarinyloxy)acetaldehyde oximes **9a-d**, with the 9-allylpurines **10a-d**. The oximes **9a-d** (84-88% yields) were prepared for first time from the corresponding substituted acetaldehydes **8a-d** [21, 22] by treatment with NH_2_OH.HCl in ethanol-water in the presence of anhydrous CH_3_COONa at 80° C for 1.5 h. The acetaldehydes **8a-d** were in turn prepared in 87-94% yields by refluxing a hydrochloric acid solution of the corresponding acetals **7a-d** for 1 h. The acetals **7a-d** were synthesized from the hydroxycoumarins **6a-d** after heating with 2-bromo-1,1-diethoxyethane and K_2_CO_3_ in DMF at 90°C for 24 h.

The reaction of 9-allylpurine **10a** with the nitrile oxide, resulted from the oxime **9a**, was carried out by the above described one-pot procedure with NCS and Et_3_N and led to the isoxazoline **11a** in 70% yield (Table **2**, entry 1). In the case of isoxazoline **11a** the coumarin moiety is connected in the 3-position of the isoxazoline ring and the purine moiety in the 5-position, differentiated from the isoxazoline **4a**. The regiochemistry of **11a** is demonstrated by HMBC experiments as there is correlation of the protons of OC*H*_2_ group with the carbon of 4-*C*H_2_ (isoxazoline) (38.3 ppm in ^13^C-NMR) and not with the 5-*C*H (isoxazoline) (78.6 ppm).

**Scheme 2:**
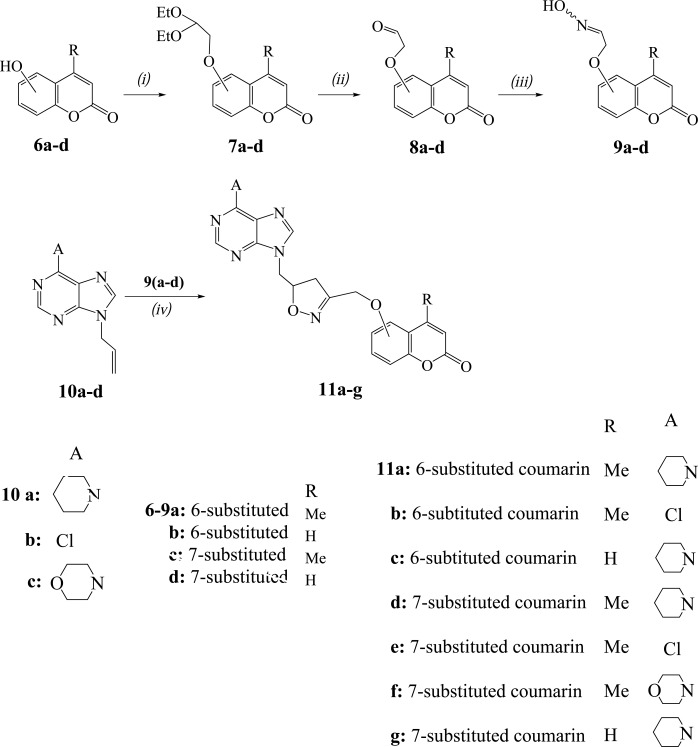
Reagents and conditions: *(i)* Anh. K_2_CO_3_ (1 equiv.), DMF (dry), 2-bromo-1,1-diethoxyethane (1 equiv.), 90°C, 24 h; *(ii)* 1N HCl, reflux, 1 h; *(iii)* NH_2_OH.HCl (1 equiv.), CH_3_COONa (anh. 0.41 equiv.), H_2_O, EtOH, 80^0^C, 1.5 h; *(iv)* DMF (dry), NCS (1.4 equiv.) in portions during 1 h, N_2_, r.t. 30 min, **10** (1 equiv.), Et_3_N (1 equiv.), r.t. 24 h.

The isoxazolines **11b,c** were isolated in 67% and 70% yields respectively from the reactions of 9-allylpurines **10b,a** with the nitrile oxides synthesized from the oximes **9a,b** (Table **2**, entries 2,3). The analogous reactions of the oxime **9c** with the purines **10a-c** in the presence of NCS and Et_3_N gave the isoxazolines **11d-f** (Table **2**, entries 4-6) (Scheme **2**). The isoxazoline **11g** was obtained from the reaction of nitrile oxide, produced from the oxime **9d**, with the purine **10a** (Table **2**, entry 7).

**Scheme 3:**
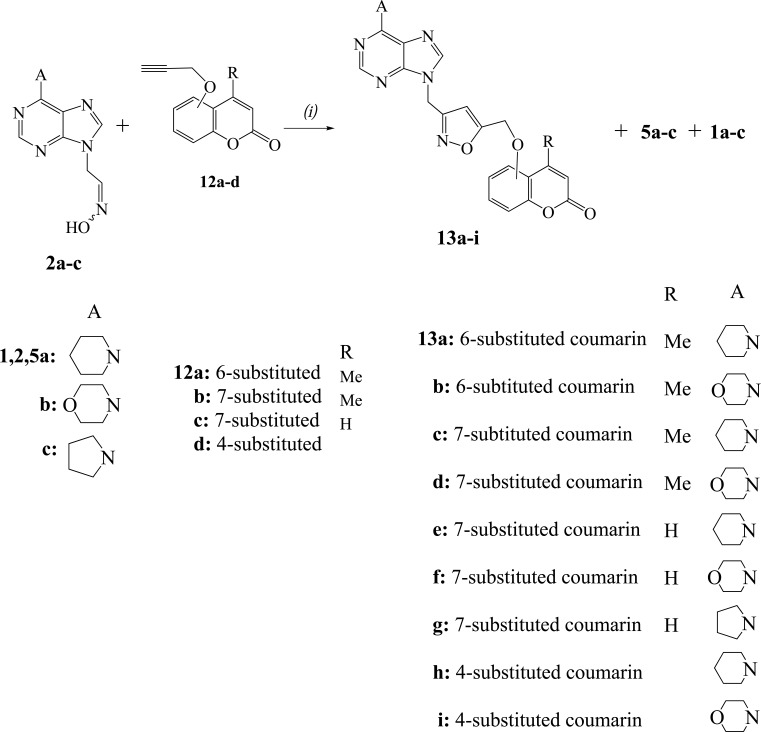
Reagents and conditions: *(i)* TFA (0.2 equiv.), **12** (1.1 equiv.), PIDA (1.1 equiv.) in MeOH, **2** (1 equiv.) in MeOH (dropwise during 1 h), r.t. 4 h.

We examined next the reactions of nitrile oxides generated from the oximes **2a-c** with the propargyloxycoumarins **12a-d** in order to obtain the hybrids **13a-i** with isoxazole ring (Scheme **3**). The reaction of propargyloxycoumarin **12a** [23] with the nitrile oxide, resulted from the oxime **2a** under the above described one-pot procedure (NCS, Et_3_N), gave the expected product **13a** only in 28% yield (Table **3**, entry 1). In order to increase the yield of this 1,3-dipolar cycloaddition reaction, we investigated the best reaction conditions using different oxidants and solvents under different temperatures (Table **3**).

PIDA as oxidant of oxime **2a**, for the formation of the corresponding nitrile oxide in MeOH under r.t. gave a little better yield for the product **13a** (37%) (Table **3**, entry 2). The method with PIDA and a catalytic [18] amount of TFA was the best with the yield for **13a** to increase (64%). By changing the temperature to 0°C or 60°C the yields for **13a** were decreased, while the amount of furoxan **5a** was increased and the hydrolysis product, aldehyde **1a**, was the major product at 60°C (Table **3**, entries 4,5). When MeOH/water was used as solvent, the yield of **13a** was a little lower (Table **3**, entry 6), while the DCM or the THF led to furoxan **5a** as the major product (Table **3**, entries 7 or 8 respectively). The increase in the amount of TFA or the PIDA gave a larger amount of the aldehyde **1a** (Table **3**, entries 9 or 10 respectively). The PIFA, which was the oxidant of choice for analogous reactions without solvent [18], led to lower yield of isoxazole **13a** (Table **3**, entry 11).

After the examination of suitable reaction conditions, the propargyloxycoumarins were reacted with the nitrile oxides generated from the oximes **2a-c** in the presence of PIDA and catalytic amount of TFA (Scheme **3**, Table **4**). The reactions of 4-methyl-6-propargyloxycoumarin (**12a**) with the oximes **2a,b** led to the isoxazoles **13a,b** respectively (Table **4**, entries 1,2). The isoxazole **13a** has the expected regiochemistry [18] as indicated by HMBC experiments. There is a correlation between the protons of NC*H*_2_ group [5.48 ppm (s, 2H)] with the carbon of 4-*C*H (isoxazole) (103.4 ppm in ^13^C-NMR) and not with the 5-*C* (isoxazole) (168.8 ppm). The isoxazoles **13c,d** were received from the reactions of 4-methyl-7-propargyloxycoumarin (**12b**) [24] with the oximes **2a,b** (Table **4**, entries 3,4). The analogous reactions of 7-propargyloxycoumarin (**12c**) [25] with the oximes **2a-c** led to the isoxazoles **13e-g** (Table **4**, entries 5-7). The isoxazoles **13h,i** were isolated from the reactions of 4-propargyloxycoumarin (**12d**) [26] with the oximes **2a,b** (Table **4**, entries 8,9).

**Scheme 4:**
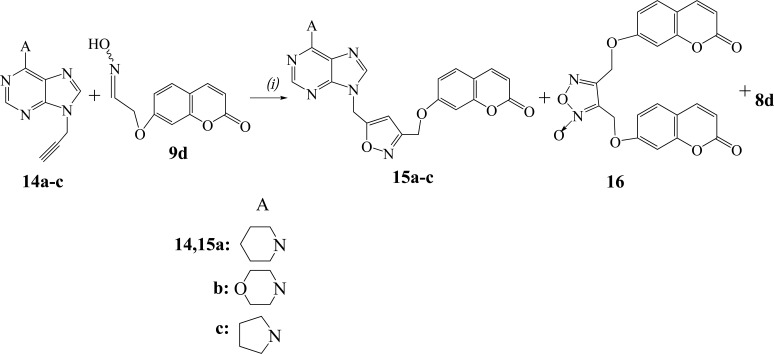
Reagents and conditions: *(i)* TFA (0.2 equiv.), **14** (1.1 equiv.), PIDA (1.1 equiv.) in MeOH, **9d** (1 equiv.) in MeOH (dropwise during 1 h), r.t. 4 h.

**Scheme 5:**
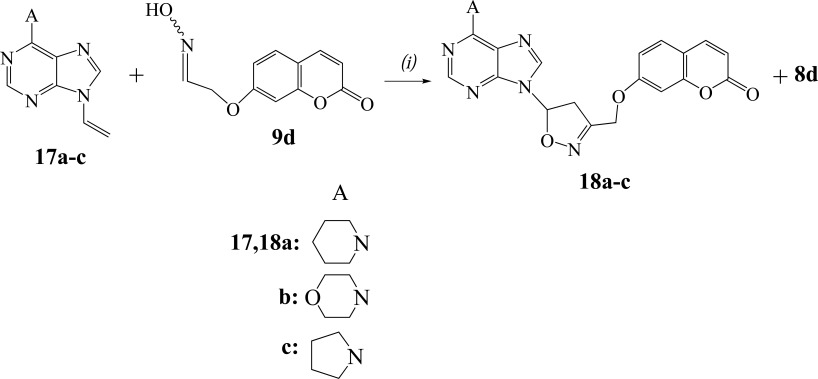
Reagents and conditions: *(i)* TFA (0.2 equiv.), **17** (1.1 equiv.), PIDA (1.1 equiv.) in MeOH, **9d** (1 equiv.) in MeOH (dropwise during 1 h), r.t. 4 h.

We studied also the reactions of oxime **9d** with the 9-propargylpurines **14a-c** (Scheme **4**) and the 9-vinylpurines **17a-c** (Scheme **5**) in the presence of PIDA and catalytic amount of TFA. From the reaction of purine **14a** the isoxazole **15a** (56%) was isolated along with the dimerization product, furoxan **16** (19%). The regiochemistry of **15a** is demonstrated by HMBC experiments as there is correlation of the protons of OC*H*_2_ group with the carbon of 4-*C*H (isoxazole) (103.1 ppm in ^13^C-NMR) and not with the 5-*C* (isoxazole) (166.8 ppm). The reactions of purines **14b,c** led to the isoxazoles **15b** (59%) and **15c** (53%) respectively, while the furoxan **16** (19% and 17%) was also formed. The above resulted isoxazoles **15a-c** were formed despite the possibility for isomerization of alkynes **14a-c** to the corresponding allenes [27].

The 9-vinylpurine **17a** [28] reacted with the oxime **9d** to give the isoxazoline **18a** (66%) (Scheme **5**). No furoxan **16** was detected in the reaction mixture. The isoxazoline **18a** has the same regiochemistry, like the others, as the protons of OC*H*_2_ group [5.06 ppm (d, 1H)/5.12 ppm (d, 1H)] in HMBC experiments are correlated with the carbon of 4-*C*H_2_ (isoxazoline) (46.9 ppm in ^13^C-NMR) and not with the 5-*C*H (isoxazoline) (84.4 ppm). The reactions of purines **17b,c** gave the isoxazolines **18b** (62%) and **18c** (60%) respectively, along with the aldehyde **8d** (15%).

### 3.2 Biological Evaluation

The formation of Reactive Oxygen Species (ROS) is a consequence of cell metabolism for aerobic organisms. Due to the extreme reactivity and tendency of ROS to initiate and participate in chain reactions, the role of antioxidants as a defense system is highly recognized. Epidemiological studies revealed the link between reactive oxygen species, inflammation, ischemia and stroke risk. A key strategy to prevent potential damage to cellular compounds such as DNA, proteins and lipids is to reduce the free radical load [29].

The compounds were studied for their antioxidant activity by the use of the stable 2,2-diphenyl-1-picrylhydrazyl radical (DPPH) at concentration 0.1mM after 20 min. A freshly prepared DPPH solution exhibits a deep purple colour with an absorption maximum at 517 nm. This purple colour generally disappears in the presence of an antioxidant. The reduction of absorbance is a measure of the free DPPH due to the action of the antioxidant. The antioxidant activity was expressed as the RA% (Reducing Activity). The RA(%) values for the tested compounds of groups **4, 11, 13, 15** and **18** at 100µM, is very low, if any with the exception of compound **4j** presenting 44% (Table **5**) in comparison to the reference drug NDGA. Within the group of derivatives **4** the presence of the morpholinyl or piperidinyl rings at position A as well as the carbonyl group at position B, hinder the interaction of the compounds to the free stable radical DPPH. It seems that compound **4j** with the combination of pyrrolidinyl ring and (CH_2_)_2_ chain (instead of a carbonyl group), present the structural features which support the interaction as well as the reducing ability and it does not face any stereochemical hindrance.

Superoxide (O_2_^-.^) anion and hydroxyl radical (^.^OH) are free radical species of potential importance. In the acidic conditions of ischemic brain, O_2_^-.^ is probably protonated to give HO_2_^-.^ species. Iron released from damaged brain cells is more likely to be readily available to catalyze the generation of OH radicals. Among the ROS, the hydroxyl (*^•^OH*) free radical is possibly the most toxic, as it reacts with a number of biological important molecules. *P*olyunsaturated fatty acids are found in high concentrations in the CNS, and are particularly vulnerable by free radicals. Thus, we tried to test the ability of our compounds to scavenge hydroxyl radicals. The competition of compounds with DMSO for HO**^.^**, generated by the Fe^3+^/ascorbic acid system, expressed as percent inhibition of formaldehyde production, was used for the evaluation of their hydroxyl radical scavenging activity In this experiment, the **13f, 13g, 13e, 13h, 15c, 15a, 13b, 13a** and **4l** showed remarkable activity at 100 μM, with values higher than the well known antioxidant trolox (Table **5**). A number of compounds like **4c, 4f, 4j, 4n, 4o, 11b, 11d, 13i,18a, 18b** did not present any activity whereas **4d, 4k, 13d** and **15d** showed lower response. Within the compounds **4a-4o** the most potent derivatives **4d** and **4j** contain a morpholinyl ring in their structure which seems to be correlated with their scavenging activity. All the derivatives of series **13** (except of **13i**) present high antioxidant activity (80-100%) which is not able to be correlated with any specific structural characteristic since all contain a coumarin, a purine and an isoxazolyl moiery. This observation is not followed within **15a-c** where both **15a** and **c** are almost equipotent whereas **15b** the morpholinyl analogue exhibits half of their activity (48%). It seems that the overall molar configuration influences the response. However, antioxidants with hydrophilic or lipophilic character are both needed to act as radical scavengers in the aqueous phase or as chain-breaking antioxidants in biological membranes.

**Anti-lipid peroxidation activity.** The water-soluble azo compound AAPH has been extensively used as a clean and controllable source of thermally produced alkyl peroxyl free radicals, through spontaneous thermal decomposition. The use of the free radical reactions initiator AAPH is recommended as more appropriate for measuring radical-scavenging activity *in vitro*, because the activity of the peroxyl radicals produced by the action of AAPH shows a greater similarity to cellular activities such as lipid peroxidation. In the AAPH assay, the highly reactive alkylperoxyl radicals are intercepted mainly by a hydrogen atom transfer (HAT) from the antioxidant. Compounds 1**3a, 13b, 13e, 15a** presented high activity whereas **4a, 4c, 4e, 4f, 11e, 13i, 13h** showed 62-86% inhibition of lipid peroxidation. The rest exhibited limited or no activity (Table **5**).

LO is the key enzyme in leukotriene biosynthesis [30]. Leukotrienes derived from the biotransformation of arachidonic acid catalyzed by 5-lipoxygenase (5-LO), are important inflammatory mediators [31] implicated in several diseases. LOs play a role in membrane lipid peroxidation by forming hydroperoxides in the lipid bilayer. Inhibitors of LO have attracted attention initially as potential agents for the treatment of inflammatory diseases. Most of the LO inhibitors are antioxidants or free radical scavengers, since lipoxygenation occurs via a carbon-centered radical [32, 33]. The evaluation of the novel coumarin hybrids against soybean lipoxygenase LO was accomplished by the UV-based enzyme assay [34].

Study of LO inhibition values demonstrates that compound **4k** provided the best activity (IC_50_ = 10μM) followed by **4n** (35μΜ), **11d** (48μΜ), **4f** (53μΜ), **13h** and **15a** (55μM), **4b** (60mM), **4l** (61mΜ), **13e** and **13g** (62.5μM), **13i** (76μΜ), **18a** (90μΜ), **15b** and **18b** (100μM). It seems that the ester **4k** is more interesting and potent hybrid compared to the corresponding ether **4a**. However, ester **4l** is almost equipotent to ether **4b**. Also the presence of a 4-methyl group enhances activity. Thus, **4k** is more potent inhibitor compared to the **4n** in which the methyl group is missing. The nature of A ring is a structural characteristic of importance. Thus, the piperidinyl derivative **4n** is more potent (35μΜ) compared to **4o** in which a morpholinyl group has replaced the piperidinyl group. The presence of pyrrolidinyl ring in compounds **4g** (10%), **11g** (no), **13g** (62.5 μΜ) is correlated with no or low activity.

Within the compounds of **11a-g** subgroup no inhibitory activities were observed. The only exception was compound **11d.** Again a piperidinyl 6-substituted coumarin derivative was found to be more potent (IC_50_ = 48μM).

Among the isoxazole coumarin derivatives **13a-i** most interesting results were given by **13h (55** μM), **13e (62.5**μM), **13g (62.5** μM), and **13i (76** μM) (**13h>13g, 13e>13i).** The most potent **13h** was a piperidinyl substituted derivative, whereas the replacement by a morpholinyl ring (**13i**) led to a decrease. Thus, the nature of the ring was implicated in the biological response. The equimolar response of **13e** and **13i** support the idea that the nature of A ring did not affect the inhibition.

Changing the attachment position of the isoxazole ring the analogues **15a-c** were taken, from which **15a** was more potent (55 μM) followed by **15b** (100 μM) and **15c** (9%). These findings follow the previous one supporting the significant role of the piperidinyl group. Also the piperidinyl derivative **18a** was found slightly more active than the **18b**.

Herein, the antilipid peroxidation activity does not go in parallel to the anti-LO activity (Table **5**). Hydroxyl scavenging activity also was not found to be correlated with the above responses.

Considering the interesting results shown in Table **5** that clearly confirm the antioxidant potential of some of the new hybrid derivatives, we found interesting to test them as ChE inhibitors. Using already described protocols for the determination of the AChE [35] inhibition we obtained the IC_50_ values shown in Table **5**. In Table **5** are only given the IC_50_ values for AChE inhibition of the more active compounds as well as the % inhibition for BChE. For comparative purposes some reference molecules have been incorporated.

Regarding the AChE inhibition (Table **5**), hybrids **4f>13e>4b>13b>13i>11b>11e>4i** showed significant eeAChE inhibition activity and they all are ethers of coumarin. The 4-, 6, or 7- position of attachment does not influence the result. For esBChE, most of the hybrids were poor inhibitors, with the exception of **13e**, one of the two most potent AChE inhibitors (**4f** and **13e**). All tested hybrids were less potent compared to the reference compound galantamine, but they retain fair AChE inhibitory activities in the low micromolar range.

For the MAO-A and B inhibition, we choose a subgroup consisted of the active anti-AChE compounds (Table **6**). Only compound **11b** showed an interesting inhibition activity IC_50_ = 9.5 μΜ against MAO-B acting as a selective agent. All the others present low (%) or not any activity at 10 μΜ. Hybrid **11b** showed very lower inhibitory potency compared with the reference clorgyline, a well-established MAO-A selective inhibitor used in the treatment of depression. Indeed, the interesting MAO-B selectivity can be considered a good starting point for a structure-based refinement, in view of the potential of MAO-B selective inhibitors as neuroprotective agents in the therapy of neurodegenerative diseases [36].

Considering the MAO inhibition data, we conclude that hybrid **11b** is a moderate, but selective inhibitor. Hybrids **4b, 4f, 4i, 11b, 11e, 13b, 13i** are potent and selective inhibitors for AChE, whereas **13e** is a dual AChE and BChE inhibitor.

## CONCLUSION

In summary, the observed antioxidant activity of the majority of the examined hybrids, allows us to propose them as templates in the design of compounds useful in treating of AD that involves reactive oxygen species (ROS). Eleven out of twenty one derivatives are potent hydroxyl radical scavengers and significant number of them inhibit *in vitro* lipid peroxidation. Compounds **4k** and **4n** present higher LO inhibitory activity among the tested derivatives. It should to be noticed that compound **13e** presents an antioxidant significant profile combining anti-LO, anti-AChE and anti-MAO-B activities. These results support the idea of a new lead compound. Overall the presented results would be possible to lead to a new multifunctional group of compounds.

## Figures and Tables

**Table 1:** Synthesis of the [3-(9*H*-purin-9-ylmethyl)-4,5-dihydroisoxazol-5-yl]methoxy-2*H*-chromen-2-ones **4a-o.**

Entry	Oxime	Alkenyloxycoumarin	Product (Yield %)
1	**2a**	**3a**	**4a** (68)
2	**2b**	**3a**	**4b** (68)
3	**2a**	**3b**	**4c** (66)
4	**2b**	**3b**	**4d** (71)
5	**2c**	**3b**	**4e** (69)
6	**2a**	**3c**	**4f** (73)
7	**2b**	**3c**	**4g** (70)
8	**2a**	**3d**	**4h** (64)
9	**2b**	**3d**	**4i** (65)
10	**2c**	**3d**	**4j** (67)
11	**2a**	**3e**	**4k** (71)*, **1a** (17%)
12	**2b**	**3e**	**4l** (68)*, **1b** (14%)
13	**2c**	**3e**	**4m** (65)*, **1c** (14%)
14	**2a**	**3f**	**4n** (65)*, **1a** (15%)
15	**2b**	**3f**	**4o** (67)*, **1b** (12%)

* By using PIDA, TFA and not NCS, Et_3_N.

**Table 2:** Synthesis of the [5-(9*H*-purin-9-ylmethyl)-4,5-dihydroisoxazol-3-yl]methoxy-2*H*-chromen-2-ones **11a-g.**

Entry	(Coumarinyloxy)acetaldehyde oxime	9-Allylpurine	Product (Yield %)
1	**9a**	**10a**	**11a** (70)
2	**9a**	**10b**	**11b** (67)
3	**9b**	**10a**	**11c** (70)
4	**9c**	**10a**	**11d** (72)
5	**9c**	**10b**	**11e** (68)
6	**9c**	**10c**	**11f** (65)
7	**9d**	**10a**	**11g** (64)

**Table 3:** Optimization of the conditions of 1,3-dipolar cycloaddition reaction of oxime **2a** (1 mmol) with the propargyloxycoumarin **12a** (1.1 mmol).

Entry	Reactants (mmol)	Solvent	T (°C)	Products (Yield %)
1	NCS (1.4), Et_3_N (1)	DMF	25	**13a** (28), **5a** (25), **2a** (45)
2	PIDA (1.1)	MeOH	25	**13a** (37), **5a** (21), **1a** (12)
3	PIDA (1.1), TFA (0.2)	MeOH	25	**13a** (64), **5a** (16), **1a** (9)
4	PIDA (1.1), TFA (0.2)	MeOH	0	**13a** (32), **5a** (45)
5	PIDA (1.1), TFA (0.2)	MeOH	60	**13a** (19), **5a** (23), **1a** (42)
6	PIDA (1.1), TFA (0.2)	MeOH/H_2_O	25	**13a** (56), **5a** (19), **1a** (15)
7	PIDA (1.1), TFA (0.2)	DCM	25	**13a** (34), **5a** (48), **1a** (12)
8	PIDA (1.1), TFA (0.2)	THF	25	**13a** (25), **5a** (52), **1a** (19)
9	PIDA (1.1), TFA (0.6)	MeOH	25	**13a** (18), **5a** (27), **1a** (48)
10	PIDA (2), TFA (0.6)	MeOH	25	**13a** (14), **5a** (31), **1a** (37)
11	PIFA (1)	MeOH	25	**13a** (45), **5a** (39), **1a** (15)

**Table 4:** Synthesis of the [3-(9*H*-purin-9-ylmethyl)isoxazol-5-yl]methoxy-2*H*-chromen-2-ones **13a-i.**

Entry	Oxime	Propargyloxycoumarin	Product (Yield %)
1	**2a**	**12a**	**13a** (64), **5a** (16), **1a** (9)
2	**2b**	**12a**	**13b** (62), **5b** (18), **1b** (9)
3	**2a**	**12b**	**13c** (57), **5a** (19), **1a** (10)
4	**2b**	**12b**	**13d** (54), **5b** (19), **1b** (12)
5	**2a**	**12c**	**13e** (56), **5a** (19), **1a** (10)
6	**2b**	**12c**	**13f** (53), **5b** (19), **1b** (11)
7	**2c**	**12c**	**13g** (53), **5c** (18), **1c** (11)
8	**2a**	**12d**	**13h** (60), **5a** (16), **1a** (10)
9	**2b**	**12d**	**13i** (58), **5b** (16), **1b** (10)

**Table 5:** *In vitro* antioxidant activity. Inhibitory activity of compounds on eeAcetylcholinesterase (eeAChE IC_50_ μΜ) and on esButyrylcholinesterase (esBuChE IC_50_ μΜ/%) of the tested compounds.

**Compds**	**RA% @ 100μΜ**	**∙ΟΗ % @ 100μΜ**	**LP % @ 100μΜ**	**%LOX @ 100μΜ or IC_50_ μΜ**	**eeAChE % inhibn. @ 10 μΜ or IC_50_ μΜ**	**esBChE % inhibn. @ 10 μΜ or IC_50_ μΜ**
**4a**	nt	nt	63	6%	nt	nt
**4b**	nt	nt	no	**60 μΜ**	**2.3 μΜ**	17%
**4c**	17	no	65	no	nt	nt
**4d**	nt	59	21	44.5%	nt	nt
**4e**	nt	nt	65	no	nt	nt
**4f**	3	no	62	**53 μΜ**	**1.4 μΜ**	36%
**4g**	nt	nt	16	10%	nt	nt
**4i**	nt	nt	54	2%	**7.7 μΜ**	15%
**4j**	44	no	32	37%	nt	nt
**4k**	12	27	57	**10 μΜ**	nt	nt
**4l**	12	90	37.5	**61 μΜ**	nt	nt
**4n**	17	no	no	**35μΜ**	nt	nt
**4o**	3	no	40	**60 μΜ**	nt	nt
**11a**	nt	nt	44	no	nt	nt
**11b**	5	no	no	no	**5.9 μΜ**	8%
**11d**	13	no	43	**48μΜ**	nt	nt
**11e**	nt	nt	63	no	**7.8 μΜ**	8%
**11f**	nt	nt	48	no	43%	14%
**11g**	nt	nt	no	no	nt	nt
**13a**	no	90	100	no	40%	23%
**13b**	no	95	90	44%	**3.1 μΜ**	13%
**13d**	no	79	no	no	nt	nt
**13e**	no	99	100	**62 μΜ**	**1.73 μΜ**	**18 μM**
**13f**	no	100	23	25%	nt	nt
**13g**	no	100	43	**62 μΜ**	nt	nt
**13h**	no	97	86	**55 μΜ**	nt	nt
**13i**	no	no	74	**76 μΜ**	**5.0 μΜ**	18%
**15a**	no	93	100	**55 μΜ**	nt	nt
**15b**	no	48	52	**100 μΜ**	nt	nt
**15c**	no	97	43	9%	nt	nt
**18a**	4	no	No	**90 μΜ**	nt	nt
**18b**	1	no	38	**100 μΜ**	nt	nt
**NDGA**	87			**5.5 μΜ**		
**Trolox**		83	76			
**Galantamine**					**0.51 μΜ**	**8.7 μΜ**

No: no activity under the reported conditions; nt: not tested

**Table 6:** *In vitro* Inhibitory activity (%) on MAO-A and on MAO-B (%).

**Compounds**	**MAO-A (%) @ 10μΜ**	**MAO-B (%) @ 10μΜ or IC_50_ μΜ**
**4b**	no	24
**4f**	no	no
**4i**	25	no
**11b**	15	**IC_50_ = 9.5 μΜ**
**11e**	no	9
**11f**	16	8
**13a**	47	33
**13b**	12	no
**13e**	18	15
**13i**	6	9
**Clorgyline**	**IC_50_ = 2.4 nM**	**IC_50_ = 2.4 μΜ**

No: no activity under the reported conditions

## SUPPORTIVE/SUPPLEMENTARY MATERIAL

The experimental data for all compounds are involved.
