# Supplementary material for: Synthesis and Biological Evaluation of Novel Hybrid Molecules Containing Purine, Coumarin and Isoxazoline or Isoxazole Moieties
Source: Open Med Chem J. 2017 Nov 30;11:196–211. doi: 10.2174/1874104501711010196 (PMC5748833; doi:10.2174/1874104501711010196)
Supplement: Supplementary file 1 [file TOMCJ-11-196_SD1.pdf]

Send Orders for Reprints to reprints@benthamscience.ae

The Open Medicinal Chemistry Journal, 2017, *11*, i-xiii

BENTHAM OPEN

CrossMark

The Open Medicinal Chemistry Journal

Supplementary Material

Content list available at: www.benthamopen.com/TOMCJ/

DOI: 10.2174/1874104501711010196

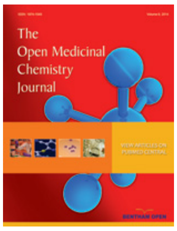

Synthesis and Biological Evaluation of Novel Hybrid Molecules Containing Purine, Coumarin and Isoxazoline or Isoxazole Moieties

Michael G. Kallitsakis[1], Angelo Carotti[2], Marco Catto[2], Aikaterini Peperidou[3], Dimitra J. Hadjipavlou-Litina[3] and Konstantinos E. Litinas[1,*]

[1]*Laboratory of Organic Chemistry, Department of Chemistry, Aristotle University of Thessaloniki, Thessaloniki 54124, Greece*

[2]*Dipartimento di Farmacia-Scienze del Farmaco, Università degli Studi di Bari "Aldo Moro", V. Orabona 4, I-70125 Bari, Italy*

[3]*Department of Pharmaceutical Chemistry, School of Pharmacy, Aristotle University of Thessaloniki, Thessaloniki 54124, Greece*

MATERIALS AND METHODS

Chemistry

Melting points were determined on a Kofler hot-stage apparatus and are uncorrected. IR spectra were obtained with a Perkin-Elmer 1310 spectrophotometer as Nujol mulls. NMR spectra were recorded on a Bruker AM 300 (300 MHz and 75 MHz for ^{1}H and ^{13}C, respectively) or on a Agilent 500/54 (DD2) (500 MHz and 125 MHz for ^{1}H and ^{13}C, respectively) using $CDCl_3$ as solvent and TMS as an internal standard. *J* values are reported in Hz. Mass spectra were determined on a Shimadzu LCMS-2010 EV system under Electrospray Ionization (ESI) conditions. Microanalyses were performed on a Perkin-Elmer 2400-II Element analyzer. Analyses indicated by the symbols of the elements or functions were within ± 0.4% of the theoretical values. Silica gel N° 60, Merck A.G. was used for column chromatography.

General Procedure for the Synthesis of (Purin-9-yl)Acetaldehyde Oximes. Synthesis of (6-morpholin-4-yl-9H-purin-9-yl)Acetaldehyde Oxime (2b)

$NH_2OH.HCl$ (0.107 g, 1.55 mmol) and anhydrous CH_3COONa (52 mg, 0.63 mmol) were added to the solution of aldehyde **1b** (0.383 g, 1.55 mmol) in H_2O/EtOH (10:4 ml) and the mixture was heated at 80°C for 2.5 h. After cooling the aqueous solution was eluted with ethyl acetate (14 x 20 ml). The organic layer was dried with anhydrous $MgSO_4$, filtered and concentrated to give **2b** (*E*/*Z*, 3:1), 0.377 g (83% yield).

(6-Morpholin-4-yl-9H-purin-9-yl)Acetaldehyde Oxime (2b)

(*E*/*Z*, 3:1) (83%), white crystals, m.p. 179-181°C (ethyl acetate); IR (Nujol): 3395, 3035, 1610, 1580, 1550 cm^{-1}; ^{1}H-NMR (DMSO-$_{d6}$, 300 MHz) (*E*/*Z*): δ = 3.83-3.95 (m, 16H), 4.43-4.61 (m, 16H), 5.14 (d, 6H, *J*=5.0 Hz), 5.21 (d, 2H, *J*=4.5 Hz), 6.94 (t, 1H, *J*=4.5 Hz), 7.60 (t, 3H, *J*=5.0 Hz), 8.27 (s, 3H), 8.30 (s, 1H), 8.50 (s, 4H); ^{13}C-NMR (DMSO-$_{d6}$, 75 MHz) (*E*): δ = 42.4, 47.2, 65.4, 117.9, 140.9, 141.9, 144.7, 146.0, 147.7; MS (ESI): *m/z* 263 $[M+H]^+$, 285 $[M+Na]^+$. Anal. Calcd (%) for $C_{11}H_{14}N_6O_2$: C, 50.38; H, 5.38; N, 32.04. Found: C, 50.32; H, 5.35; N, 32.01.

(6-Pyrrolidin-1-yl-9H-purin-9-yl)Acetaldehyde Oxime (2c)

(*E*/*Z*, 3:1) (80%), white crystals, m.p. 215-216°C (ethyl acetate); IR (Nujol): 3380, 3060, 1610, 1545 cm^{-1}; ^{1}H-NMR ($CDCl_3$, 300 MHz) (*E*/*Z*): δ = 1.96-2.13 (m, 16H), 3.76-4.31 (m, 16H), 4.95 (d, 6H, *J*=5.2 Hz), 5.14 (d, 2H, *J*=4.4 Hz),

6.93 (t, 1H, *J*=4.4 Hz), 7.62 (t, 3H, *J*=5.2 Hz), 7.78 (s, 3H), 7.80 (s, 1H), 8.40 (s, 4H); ^{13}C-NMR (DMSO-$_{d6}$, 75 MHz) (*E*): δ = 29.2, 42.7, 50.0, 51.5, 119.0, 142.3, 142.7, 144.5, 145.8, 148.5; MS (ESI): *m/z* 247 $[M+H]^+$; Anal. Calcd (%) for $C_{11}H_{14}N_6O$: C, 53.65; H, 5.73; N, 34.13. Found: C, 53.59; H, 5.71; N, 34.02.

Synthesis of 2-oxo-2H-chromen-7-yl Acrylate (3f)

Anhydrous K_2CO_3 (0.392 g, 2.84 mmol) and acryloyl chloride (0.15 ml, 0.257 g, 2.84 mmol) were added to the solution of 7-hydroxycoumarin (0.46 g, 2.84 mmol) in DMF (15 ml). The resulted mixture was heated at 90°C for 24 h. The hot mixture was filtered and washed in the filter with DCM. The filtrates were evaporated and the residue was separated by column chromatography [hexane/ethyl acetate (4:1)] to give compound **3f**, 0.521 g (85% yield), white crystals, m.p. 130-132°C (DCM); IR (KBr): 3037, 1716, 1708, 1596 cm^{-1}; ^{1}H-NMR ($CDCl_3$, 300 MHz) δ 6.07 (d, 1H, *J*=10.5 Hz), 6.32 (dd, 1H, J_1=10.5 Hz, J_2=17.3 Hz), 6.39 (d, 1H, *J*=9.6 Hz), 6.64 (d, 1H, *J*=17.3 Hz), 7.09 (d, 1H, *J*=8.4 Hz), 7.16 (s, 1H), 7.50 (d, 1H, *J*=8.4 Hz), 7.69 (d, 1H, *J*=9.6 Hz); ^{13}C-NMR ($CDCl_3$, 75 MHz) δ 110.4, 116.1, 116.7, 118.4, 127.3, 128.6, 133.7, 142.9, 153.1, 154.7, 160.3, 163.8; MS (ESI): *m/z* 217 $[M+H]^+$; Anal. Calcd (%) for $C_{12}H_8O_4$: C, 66.67; H, 3.73. Found: C, 66.71; H, 3.68.

General Procedure. 1,3-Dipolar Cycloaddition Reactions of (purin-9-yl)Acetaldehyde Oximes with Alkenyloxycoumarins. Synthesis of 4-methyl-6-({3-[(6-piperidin-1-yl-9H-purin-9-yl)methyl]-4,5-dihydroisoxazol-5-yl}methoxy)-2H-chromen-2-one (4a).

In the solution of oxime **2a** (41 mg, 0.16 mmol) in dry DMF (5 ml) NCS (32 mg, 0.22 mmol) was added under stirring in portions during 1 h. The resulted mixture was stirred for 30 min. The allyloxycoumarin **3a** (32 mg, 0.16 mmol) and Et_3N (0.03 ml, 16 mg, 0.16 mmol) were then added and the mixture was stirred at r.t. for 24 h under N_2 atmosphere. The mixture was filtered, the solid was washed with DCM and the filtrate was evaporated. The residue was chromatographed in a column [hexane/ethyl acetate (2:1)] and gave after the elution of starting coumarin **3a** (5 mg, 16%) the isoxazoline **4a**, 51 mg, (68% yield).

4-Methyl-6-({3-[(6-piperidin-1-yl-9H-purin-9-yl)methyl]-4,5-dihydroisoxazol-5-yl}methoxy)-2H-chromen-2-one (4a)

White crystals, m.p.180-182°C (ethyl acetate); IR (Nujol): 3020, 1715, 1620, 1570 cm^{-1}; ^{1}H-NMR ($CDCl_3$, 500 MHz) δ 1.63-1.80 (m, 6H), 2.40 (d, 3H, *J*=1.2 Hz), 2.98 (dd, 1H, J_1=7.2 Hz, J_2=17.1 Hz), 3.09 (dd, 1H, J_1=11.2 Hz, J_2=17.1 Hz), 4.05 (d, 2H, *J*=4.3 Hz), 4.17-4.29 (m, 4H), 4.93-5.05 (m, 1H), 5.15 (s, 2H), 6.29 (d, 1H, *J*=1.2 Hz), 6.99-7.05 (m, 2H), 7.23 (d, 1H, *J*=8.8 Hz), 7.80 (s, 1H), 8.32 (s, 1H); ^{13}C-NMR ($CDCl_3$, 125 MHz) δ 18.6, 24.7, 26.1, 37.5, 39.8, 45.6, 69.7, 78.6, 109.3, 115.7, 118.1, 119.2, 119.6, 120.6, 137.6, 150.6, 151.7, 152.6, 153.9, 154.3, 154.8, 160.7, 161.2; MS (ESI): *m/z* 475 $[M+H]^+$, 497 ;$[M+Na]^+$; Anal. Calcd (%) for $C_{25}H_{26}N_6O_4$: C, 63.28; H, 5.52; N, 17.71. Found: C, 63.17; H, 5.47; N, 17.86.

4-Methyl-6-({3-[(6-morpholin-4-yl-9H-purin-9-yl)methyl]-4,5-dihydroisoxazol-5-yl}methoxy)-2H-chromen-2-one (4b)

White crystals (68%), m.p.184-186°C (ethyl acetate); IR (KBr): 3014, 2912, 2846, 1723, 1632, 1567, 1508 cm^{-1}; ^{1}H-NMR ($CDCl_3$, 300 MHz) δ 2.40 (s, 3H), 3.01 (dd, 1H, J_1=7.2 Hz, J_2=17.9 Hz), 3.14 (dd, 1H, J_1=11.3 Hz, J_2=17.9 Hz), 3.77-3.87 (m, 4H), 4.08 (d, 2H, *J*=4.4 Hz), 4.28-4.38 (m, 4H), 4.94-5.05 (m, 1H), 5.20 (s, 2H), 6.29 (s, 1H), 6.99-7.07 (m, 2H), 7.24 (d, 1H, *J*=8.9 Hz), 7.82 (s, 1H), 8.36 (s, 1H); ^{13}C-NMR ($CDCl_3$, 75 MHz) δ 18.7, 37.7, 43.5, 46.4, 67.0, 69.6, 79.5, 109.3, 115.8, 118.1, 119.3, 119.6, 120.6, 138.8, 148.5, 150.8, 151.8, 154.0, 154.8, 155.9, 160.7, 161.4; MS (ESI): *m/z* 477 $[M+H]^+$; Anal. Calcd (%) for $C_{24}H_{24}N_6O_5$: C, 60.50; H, 5.08; N, 17.64. Found: C, 60.62; H, 5.19; N, 17.49.

54-Methyl-7-({3-[(6-piperidin-1-yl-9H-purin-9-yl)methyl]-4,5-dihydroisoxazol-5-yl}methoxy)-2H-chromen-2-one (4c)

White crystals (66%), m.p.186-187°C (ethyl acetate); IR (Nujol): 3060, 1720, 1590, 1545 cm^{-1}; ^{1}H-NMR ($CDCl_3$, 300 MHz) δ 1.63-1.78 (m, 6H), 2.39 (s, 3H), 2.97 (dd, 1H, J_1=6.7 Hz, J_2=17.5 Hz), 3.12 (dd, 1H, J_1=11.4 Hz, J_2=17.5 Hz), 4.07 (d, 2H, *J*=4.7 Hz), 4.16-4.33 (m, 4H), 4.94-5.05 (m, 1H), 6.14 (s, 1H), 6.65-6.83 (m, 2H), 7.47 (d, 1H, *J*=8.5 Hz), 7.81 (s, 1H), 8.33 (s, 1H); ^{13}C-NMR ($CDCl_3$, 75 MHz) δ 18.7, 24.8, 26.2, 37.6, 39.8, 44.5, 69.0, 78.9, 101.7, 112.5,

112.8, 114.2, 120.1, 124.9, 125.7, 138.0, 150.2, 152.4, 152.6, 154.3, 155.1, 161.1, 161.2; MS (ESI): *m/z* 475 $[M+H]^+$; Anal. Calcd (%) for $C_{25}H_{26}N_6O_4$: C, 63.28; H, 5.52; N, 17.71. Found: C, 63.34; H, 5.46; N, 17.63.

4-Methyl-7-({3-[(6-morpholin-4-yl-9H-purin-9-yl)methyl]-4,5-dihydroisoxazol-5-yl}methoxy)-2H-chromen-2-one (4d)

White crystals (71%), m.p.194-196°C (ethyl acetate); IR (KBr): 3067, 2946, 1723, 1634, 1592 cm^{-1}; ^{1}H-NMR ($CDCl_3$, 300 MHz) δ 2.38 (s, 3H), 2.97 (dd, 1H, J_1=7.6 Hz, J_2=17.8 Hz), 3.12 (dd, 1H, J_1=11.6 Hz, J_2=17.8 Hz), 3.79-3.86 (m, 4H), 4.08 (d, 2H, *J*=4.4 Hz), 4.28-4.36 (m, 4H), 4.93-5.04 (m, 1H), 5.16 (s, 2H), 6.13 (s, 1H), 6.72-6.82 (m, 2H), 7.47 (d, 1H, *J*=8.5 Hz), 7.80 (s, 1H), 8.36 (s, 1H); ^{13}C-NMR ($CDCl_3$, 75 MHz) δ 18.6, 37.8, 40.8, 46.7, 67.0, 69.1, 79.3, 102.2, 112.3, 112.6, 114.4, 119.6, 122.2, 125.7, 139.1, 149.8, 152.3, 153.9, 155.3, 157.1, 161.0, 161.3; MS (ESI): *m/z* 477 $[M+H]^+$; Anal. Calcd (%) for $C_{24}H_{24}N_6O_5$: C, 60.50; H, 5.08; N, 17.64. Found: C, 60.64; H, 4.94; N, 17.69.

4-Methyl-7-({3-[(6-pyrrolidin-1-yl-9H-purin-9-yl)methyl]-4,5-dihydroisoxazol-5-yl}methoxy)-2H-chromen-2-one (4e)

White crystals (69%), m.p.190-192°C (ethyl acetate); IR Nujol): 3040, 1705, 1575 cm^{-1}; ^{1}H-NMR ($CDCl_3$, 300 MHz) δ 1.98-2.07 (m, 4H), 2.38 (d, 3H, *J*=1.1 Hz), 2.96 (dd, 1H, J_1=6.8 Hz, J_2=17.7 Hz), 3.20 (dd, 1H, J_1=8.7 Hz, J_2=17.7 Hz), 3.69-4.25 (m, 4H), 4.07 (d, 2H, *J*=4.5 Hz), 4.93-5.05 (m, 1H), 5.16 (s, 2H), 6.14 (d, 1H, *J*=1.1 Hz), 6.74 (d, 1H, *J*=2.5 Hz), 6.80 (dd, 1H, J_1=2.5 Hz, J_2=8.8 Hz), 7.47 (d, 1H, *J*=8.8 Hz), 7.81 (s, 1H), 8.35 (s, 1H); ^{13}C-NMR ($CDCl_3$, 75 MHz) δ 18.6, 31.8, 39.8, 41.8, 47.9, 48.9, 69.0, 78.9, 101.9, 112.3, 112.5, 114.3, 119.9, 125.6, 126.0, 138.5, 150.0, 152.2, 152.7, 154.2, 155.2, 160.1, 161.2; MS (ESI): *m/z* 461 $[M+H]^{+\cdot}$, 483 $[M+Na]^{+\cdot}$. Anal. Calcd (%) for $C_{24}H_{24}N_6O_4$: C, 62.60; H, 5.25; N, 18.25. Found: C, 62.73; H, 5.14; N, 18.16.

4-({3-[(6-piperidin-1-yl-9H-purin-9-yl)methyl]-4,5-dihydroisoxazol-5-yl}methoxy)-2H-chromen-2-one (4f)

White crystals (73%), m.p.142-143°C (ethyl acetate); IR KBr): 3068, 2942, 1705, 1586 cm^{-1}; ^{1}H-NMR ($CDCl_3$, 500 MHz) δ 1.68-1.81 (m, 6H), 3.09 (dd, 1H, J_1=6.2 Hz, J_2=17.2 Hz), 3.29 (dd, 1H, J_1=10.5 Hz, J_2=17.2 Hz), 4.14 (d, 1H, *J*=4.1 Hz), 4.17 (d, 1H, *J*=3.1 Hz), 4.21-4.38 (m, 4H), 5.06-5.17 (m, 1H), 5.30 (s, 2H), 5.65 (s, 1H), 7.22-7.36 (m, 2H), 7.53 (d, 1H, *J*=7.5 Hz), 7.63 (d, 1H, *J*=8.5 Hz), 7.85 (s, 1H), 8.39 (s, 1H); ^{13}C-NMR ($CDCl_3$, 125 MHz) δ 24.7, 26.1, 37.6, 39.8, 45.5, 69.3, 78.1, 90.9, 115.1, 116.8, 119.4, 122.8, 124.1, 132.6, 137.5, 150.4, 152.6, 153.2, 153.6, 154.3, 162.5, 165.0; MS (ESI): *m/z* 461 $[M+H]^+$; Anal. Calcd (%) for $C_{24}H_{24}N_6O_4$: C, 62.60; H, 5.25; N, 18.25. Found: C, 62.81; H, 5.32; N, 18.09.

4-({3-[(6-morpholin-4-yl-9H-purin-9-yl)methyl]-4,5-dihydroisoxazol-5-yl}methoxy)-2H-chromen-2-one (4g)

White crystals (70%), m.p.158-160°C (ethyl acetate); IR (Nujol): 3030, 1720,1570 cm^{-1}; ^{1}H-NMR ($CDCl_3$, 300 MHz) δ 3.01 (dd, 1H, J_1=7.4 Hz, J_2=17.6 Hz), 3.21 (dd, 1H, J_1=11.4 Hz, J_2=17.6 Hz), 3.81-3.84 (m, 4H), 4.16 (d, 1H, *J*=3.7 Hz), 4.18 (d, 1H, *J*=3.1 Hz), 4.26-4.37 (m, 4H), 5.03-5.14 (m, 1H), 5.20 (s, 2H), 5.64 (s, 1H), 7.22-7.34 (m, 2H), 7.54 (dd, 1H, J_1=1.2 Hz, J_2=8.5 Hz), 7.59 (dd, 1H, J_1=1.2 Hz, J_2=8.5 Hz), 7.83 (s, 1H), 8.34 (s, 1H); ^{13}C-NMR ($CDCl_3$, 75 MHz) δ 37.7, 39.8, 45.8, 67.1, 69.4, 78.3, 91.2, 115.3, 116.9, 119.8, 122.8, 124.1, 132.7, 138.1, 150.9, 152.7, 153.4, 154.0, 154.1, 162.3, 164.5; MS (ESI): *m/z* 463 $[M+H]^+$, 485 $[M+Na]^+$; Anal. Calcd (%) for $C_{23}H_{22}N_6O_5$: C, 61.22; H, 5.34; N, 17.13. Found: C, 61.38; H, 5.21; N, 17.28.

4-Methyl-6-(2-{3-[(6-piperidin-1-yl-9H-purin-9-yl)methyl]-4,5-dihydroisoxazol-5-yl}ethoxy)-2H-chromen-2-one (4h)

White crystals (64%), m.p.168-169°C (ethyl acetate); IR (KBr): 3012, 2931, 1708, 1624, 1563 cm^{-1}; ^{1}H-NMR ($CDCl_3$, 500 MHz) δ 1.67-1.78 (m, 6H), 1.97-2.16 (m, 2H), 2.39 (s, 3H), 2.75 (dd, 1H, J_1=7.2 Hz, J_2=17.9 Hz), 3.07 (dd, 1H, J_1=10.6 Hz, J_2=17.9 Hz), 4.05-4.17 (m, 2H), 4.23-4.34 (m, 4H), 4.83-4.92 (m, 1H), 5.16 (s, 2H), 6.18 (s, 1H), 6.71-6.84 (m, 2H), 7.49 (d, 1H, *J*=8.7 Hz), 7.76 (s, 1H), 8.35 (s, 1H); ^{13}C-NMR ($CDCl_3$, 125 MHz) δ 18.7, 24.6, 26.2, 29.3, 39.7, 42.6, 46.4, 63.8, 78.9, 101.9, 112.4, 113.7, 114.3, 119.7, 124.3, 137.8, 149.8, 151.8, 152.2, 153.7, 154.1, 154.8, 161.0, 161.9; MS (ESI): *m/z* 489 $[M+H]^+$; Anal. Calcd (%) for $C_{26}H_{28}N_6O_4$: C, 63.92; H, 5.78; N, 17.20. Found: C, 64.04; H, 5.64; N, 17.06.

4-Methyl-6-(2-{3-[(6-morpholin-4-yl-9H-purin-9-yl)methyl]-4,5-dihydroisoxazol-5-yl}ethoxy)-2H-chromen-2-one (4i)

White crystals (65%), m.p.161-163°C (ethyl acetate); IR (Nujol): 3060, 1725, 1635, 1580 cm^{-1}; ^{1}H-NMR ($CDCl_3$, 300 MHz) δ 1.92-2.11 (m, 2H), 2.38 (s, 3H), 2.72 (dd, 1H, J_1=7.4 Hz, J_2=17.5 Hz), 3.07 (dd, 1H, J_1=10.8 Hz, J_2=17.5 Hz), 3.77-3.88 (m, 4H), 4.01-4.13 (m, 2H), 4.15 (d, 1H, *J*=6.0 Hz), 4.24-4.37 (m, 4H), 4.81-4.94 (m, 1H), 5.13 (s, 2H), 6.13 (s, 1H), 6.69-6.83 (m, 2H), 7.47 (d, 1H, *J*=8.6 Hz), 7.79 (s, 1H), 8.36 (s, 1H); ^{13}C-NMR ($CDCl_3$, 75 MHz) δ 18.6, 29.3, 36.5, 40.7, 45.7, 64.7, 67.1, 78.5, 101.8, 112.2, 113.9, 116.2, 119.8, 125.6, 138.1, 150.9, 152.3, 152.8, 154.1, 154.4, 155.3, 161.1, 161.6; MS (ESI): *m/z* 491 $[M+H]^+$, 513 $[M+Na]^+$; Anal. Calcd (%) for $C_{25}H_{26}N_6O_5$: C, 59.73; H, 4.79; N, 18.17. Found: C, 59.82; H, 4.72; N, 18.28.

4-Methyl-6-(2-{3-[(6-pyrrolidin-1-yl-9H-purin-9-yl)methyl]-4,5-dihydroisoxazol-5-yl}ethoxy)-2H-chromen-2-one (4j)

White crystals (67%), m.p.178-180°C (ethyl acetate); IR (KBr): 3025, 2921, 2857, 1723, 1612, 1583 cm^{-1}; ^{1}H-NMR ($CDCl_3$, 500 MHz) δ 1.91-2.18 (m, 6H), 2.40 (s, 3H), 2.72 (dd, 1H, J_1=6.8 Hz, J_2=17.5 Hz), 3.06 (dd, 1H, J_1=10.9 Hz, J_2=17.5 Hz), 3.81-4.29 (m, 6H), 4.86-4.98 (m, 1H), 5.21 (s, 2H), 6.19 (s, 1H), 6.73-6.82 (m, 2H), 7.46 (d, 1H, *J*=8.9 Hz), 7.78 (s, 1H), 8.37 (s, 1H); ^{13}C-NMR ($CDCl_3$, 125 MHz) δ 18.7, 29.8, 31.2, 39.8, 41.4, 47.6, 48.6, 64.2, 78.1, 101.6, 112.8, 113.2, 113.8, 120.1, 124.8, 126.0, 138.4, 150.6, 151.3, 151.8, 154.7, 155.2, 160.8, 161.3; MS (ESI): *m/z* 475 $[M+H]^{+}$. Anal. Calcd (%) for $C_{25}H_{26}N_6O_4$: C, 63.28; H, 5.52; N, 17.71. Found: C, 63.42; H, 5.41; N, 17.58.

General procedure. 1,3-Dipolar cycloaddition reactions of (purin-9-yl)acetaldehyde oximes with coumarinyl acrylates. Synthesis of 4-methyl-2-oxo-2H-chromen-6-yl 3-[(6-piperidin-1-yl-9H-purin-9-yl)methyl]-4,5-dihydroisoxazole-5-carboxylate (4k).

A solution of oxime **2a** (62 mg, 0.24 mmol) in methanol (2 ml) was added dropwise during 1.5 h at r.t. in a mixture of acrylate **3e** (60 mg, 0.26 mmol), PIDA (84 mg, 0.26 mmol) and TFA (4 μl, 5.7 mg, 0.05 mmol) in methanol (3 ml). The mixture was stirred for 4 h at r.t.. Then, the solvent was evaporated and the residue was separated by column chromatography [hexane/ethyl acetate (2:1)] to give the aldehyde **1a** (10 mg, 17%) followed by the isoxazoline **4k**, 83 mg (71% yield). This reaction between the oxime **2a** and acrylate **3e** in the presence of NCS and Et_3N gave the isoxazoline **4k** (34% yield) along with the furoxan **5a** (27%).

4-Methyl-2-oxo-2H-chromen-6-yl 3-[(6-piperidin-1-yl-9H-purin-9-yl)methyl]-4,5-dihydroisoxazole-5-carboxylate (4k),

White crystals, m.p.179-181°C (ethyl acetate); IR (KBr): 3027, 2912, 2834, 1719, 1702, 1621, 1589 cm^{-1}; ^{1}H-NMR ($CDCl_3$, 500 MHz) δ 1.69-1.77 (m, 6H), 2.41 (s, 3H), 3.38-3.47 (m, 2H), 4.22-4.34 (m, 4H), 5.25 (s, 2H), 5.29 (dd, 1H, J_1=6.9 Hz, J_2=10.7 Hz), 6.32 (s, 1H), 7.21-7.28 (m, 2H), 7.36 (d, 1H, *J*=8.7 Hz), 7.81 (s, 1H), 8.35 (s, 1H); ^{13}C-NMR ($CDCl_3$, 125 MHz) δ 18.7, 24.5, 26.1, 39.4, 39.8, 46.6, 78.2, 116.0, 117.0, 118.2, 119.3, 120.7, 124.7, 137.8, 146.0, 149.1, 150.7, 151.3, 151.5, 153.3, 154.1, 160.1, 167.9; MS (ESI): *m/z* 489 $[M+H]^+$; Anal. Calcd (%) for $C_{25}H_{24}N_6O_5$: C, 61.47; H, 4.95; N, 17.20. Found: C, 61.58; H, 4.92; N, 17.12.

4-Methyl-2-oxo-2H-chromen-6-yl 3-[(6-morpholin-4-yl-9H-purin-9-yl)methyl]-4,5-dihydroisoxazole-5-carboxylate (4l)

White crystals (68%), m.p.188-189°C (ethyl acetate); IR (KBr): 3034, 2917, 1723, 1712, 1592 cm^{-1}; ^{1}H-NMR ($CDCl_3$, 500 MHz) δ 2.40 (s, 3H), 3.31-3.49 (m, 2H), 3.79-3.86 (m, 4H), 4.24-4.37 (m, 4H), 5.23 (s, 2H), 5.28 (dd, 1H, J_1=7.1 Hz, J_2=10.9 Hz), 6.33 (s, 1H), 7.22-7.28 (m, 2H), 7.34 (d, 1H, *J*=8.3 Hz), 7.81 (s, 1H), 8.37 (s, 1H); ^{13}C-NMR ($CDCl_3$, 125 MHz) δ 18.7, 39.5, 40.7, 46.7, 67.0, 78.5, 116.1, 117.0, 118.3, 119.6, 120.9, 124.7, 138.9, 146.2, 149.5, 150.0, 151.4, 151.6, 153.1, 153.9, 160.1, 167.9; MS (ESI): *m/z* 491 $[M+H]^+$; Anal. Calcd (%) for $C_{24}H_{22}N_6O_6$: C, 58.77; H, 4.52; N, 17.13. Found: C, 58.95; H, 4.29; N, 17.02.

4-Methyl-2-oxo-2H-chromen-6-yl 3-[(6-pyrrolidin-1-yl-9H-purin-9-yl)methyl]-4,5-dihydroisoxazole-5-carboxylate (4m)

White crystals (65%), m.p.192-193°C (ethyl acetate); IR (KBr): 3046, 2918, 2841, 1721, 1716, 1609, 1584 cm^{-1}; ^{1}H-NMR ($CDCl_3$, 300 MHz) δ 1.92-2.08 (m, 4H), 2.41 (s, 3H), 3.35-3.48 (m, 2H), 3.81-4.21 (m, 4H), 5.23 (s, 2H), 5.24

(dd, 1H, J_1=6.9 Hz, J_2=10.2 Hz), 6.31 (s, 1H), 7.21-7.27 (m, 2H), 7.32 (d, 1H, *J*=8.5 Hz), 7.79 (s, 1H), 8.38 (s, 1H); ^{13}C-NMR ($CDCl_3$, 75 MHz) δ 18.8, 31.9, 39.0, 42.5, 47.4, 78.6, 115.8, 117.5, 117.8, 119.8, 120.2, 124.5, 138.1, 145.8, 149.4, 150.2, 151.0, 151.4, 153.6, 154.5, 160.3, 167.8; MS (ESI): *m/z* 475 $[M+H]^+$; Anal. Calcd (%) for $C_{24}H_{22}N_6O_5$: C, 60.75; H, 4.67; N, 17.71. Found: C, 60.96; H, 4.56; N, 17.53.

2-Oxo-2H-chromen-6-yl 3-[(6-piperidin-1-yl-9H-purin-9-yl)methyl]-4,5-dihydroisoxazole-5-carboxylate (4n)

White crystals (65%), m.p.175-177°C (ethyl acetate); IR (KBr): 3012, 2918, 1729, 1703, 1592 cm^{-1}; ^{1}H-NMR ($CDCl_3$, 300 MHz) δ 1.67-1.78 (m, 6H), 3.36-3.43 (m, 2H), 4.21-4.34 (m, 4H), 5.21 (s, 2H), 5.27 (dd, 1H, J_1=6.7 Hz, J_2=10.4 Hz), 6.48 (d, 1H, *J*=9.6 Hz), 7.23-7.31 (m, 2H), 7.36 (d, 1H, *J*=8.8 Hz), 7.68 (d, 1H, *J*=9.6 Hz), 7.81 (s, 1H), 8.36 (s, 1H); ^{13}C-NMR ($CDCl_3$, 75 MHz) δ 24.6, 26.2, 39.4, 40.2, 46.2, 78.1, 112.6, 117.8, 118.1, 119.3, 120.0, 124.8, 137.7, 138.1, 142.6, 150.2, 151.4, 151.9, 153.9, 154.2, 160.2, 167.9; MS (ESI): *m/z* 475 $[M+H]^+$; Anal. Calcd (%) for $C_{24}H_{22}N_6O_5$: C, 60.75; H, 4.67; N, 17.71. Found: C, 60.82; H, 4.62; N, 17.58.

2-Oxo-2H-chromen-6-yl 3-[(6-morpholin-4-yl-9H-purin-9-yl)methyl]-4,5-dihydroisoxazole-5-carboxylate (4o)

White crystals (67%), m.p.184-185°C (ethyl acetate); IR (KBr): 3038, 2921, 2837, 1726, 1717, 1612, 1576 cm^{-1}; ^{1}H-NMR ($CDCl_3$, 500 MHz) δ 3.32-3.43 (m, 2H), 3.76-3.87 (m, 4H), 4.26-4.37 (m, 4H), 5.18 (s, 2H), 5.25 (dd, 1H, J_1=6.4 Hz, J_2=10.8 Hz), 6.45 (d, 1H, *J*=9.5 Hz), 7.26-7.32 (m, 2H), 7.38 (d, 1H, *J*=8.4 Hz), 7.64 (d, 1H, *J*=9.5 Hz), 7.79 (s, 1H), 8.34 (s, 1H); ^{13}C-NMR ($CDCl_3$, 125 MHz) δ 39.2, 40.8, 44.2, 64.7, 78.8, 114.2, 117.1, 117.9, 119.2, 120.3, 125.6, 135.4, 137.9, 144.2, 149.8, 151.2, 151.5, 153.2, 153.9, 160.1, 167.4; MS (ESI): *m/z* 477 $[M+H]^+$; Anal. Calcd (%) for $C_{23}H_{20}N_6O_6$: C, 57.98; H, 4.23; N, 17.64. Found: C, 58.06; H, 4.16; N, 17.56.

General Procedure for the Synthesis of Acetals 7a-d. Synthesis of 6-(2,2-diethoxyethoxy)-4-methyl-2H-chromene-2-one (7a).

Anhydrous K_2CO_3 (0.4 g, 2.84 mmol) was added to the solution of hydroxycoumarin **6a** (0.5 g, 2.84 mmol) in DMF (10 ml) followed by 2-bromo-1,1-diethoxyethane (0.42 ml, 0.559 g, 2.84 mmol). The resulted mixture was heated at 90°C for 24 h, filtered while hot and the solid was washed with DCM. The filtrate was evaporated and the residue was separated by column chromatography [hexane/ethyl acetate (3:1)] to give compound **7a**, 0.711 g (86% yield).

6-(2,2-Diethoxyethoxy)-4-methyl-2H-chromene-2-one (7a)

White crystals, m.p.66-68°C (DCM); IR (KBr): 3068, 2957, 2853, 1712, 1584 cm^{-1}; ^{1}H-NMR ($CDCl_3$, 300 MHz) δ 1.26 (t, 6H, *J*=7.0 Hz), 2.40 (s, 3H), 3.65 (dq, 2H, J_1=7.0 Hz, J_2=9.4 Hz), 3.79 (dq, 2H, J_1=7.0 Hz, J_2=9.4 Hz), 4.05 (d, 2H, *J*=5.1 Hz), 4.85 (t, 1H, *J*=5.1 Hz), 6.29 (s, 1H), 7.08 (d, 1H, *J*=2.8 Hz), 7.13 (dd, 1H, J_1=2.8 Hz, J_2=8.9 Hz), 7.25 (d, 1H, *J*=8.9 Hz); ^{13}C-NMR ($CDCl_3$, 75 MHz) δ 15.3, 18.6, 62.8, 69.5, 100.6, 108.9, 115.5, 117.9, 119.4, 120.5, 148.2, 151.8, 155.1, 160.8; MS (ESI): *m/z* 293 $[M+H]^+$; Anal. Calcd (%) for $C_{16}H_{20}O_5$: C, 65.74; H, 6.90. Found: C, 65.87; H, 6.83.

6-(2,2-Diethoxyethoxy)-2H-chromene-2-one (7b)

White crystals (81%), m.p.53-54°C (DCM); IR (KBr): 3036, 2936, 2865, 1704, 1562 cm^{-1}; ^{1}H-NMR ($CDCl_3$, 300 MHz) δ 1.14 (t, 6H, *J*=7.0 Hz), 3.48-3.60 (m, 2H), 3.62-3.72 (m, 2H), 3.92 (d, 2H, *J*=5.0 Hz), 4.73 (t, 1H, *J*=5.0 Hz), 6.29 (d, 1H, *J*=9.5 Hz), 6.87 (d, 1H, *J*=2.4 Hz), 7.02 (dd, 1H, J_1=2.4 Hz, J_2=9.0 Hz), 7.11 (d, 1H, *J*=9.0 Hz), 7.56 (d, 1H, *J*=9.5 Hz); ^{13}C-NMR ($CDCl_3$, 75 MHz) δ 15.5, 63.0, 69.6, 100.7, 111.5, 117.2, 117.9, 119.4, 120.3, 143.4, 148.8, 155.4, 160.9; MS (ESI): *m/z* 279 $[M+H]^+$; Anal. Calcd (%) for $C_{15}H_{18}O_5$: C, 64.74; H, 6.52. Found: C, 64.68; H, 6.63.

7-(2,2-Diethoxyethoxy)-4-methyl-2H-chromene-2-one (7c)

White crystals (79%), m.p.59-61°C (DCM); IR (KBr): 3046, 2922, 2854, 1718, 1572 cm^{-1}; ^{1}H-NMR ($CDCl_3$, 300 MHz) δ 1.26 (t, 6H, *J*=7.6 Hz), 2.36 (s, 3H), 3.58-3.71 (m, 2H), 3.72-3.86 (m, 2H), 4.04 (d, 2H, *J*=5.1 Hz), 4.86 (t, 1H, *J*=5.1 Hz), 6.06 (s, 1H), 6.75 (d, 1H, *J*=2.0 Hz), 6.86 (dd, 1H, J_1=2.0 Hz, J_2=9.6 Hz), 7.45 (d, 1H, *J*=9.6 Hz); ^{13}C-NMR ($CDCl_3$, 75 MHz) δ 15.2, 18.4, 62.8, 69.0, 100.2, 101.8, 112.0, 112.4, 113.8, 125.5, 152.3, 155.1, 161.0, 161.6; MS (ESI): *m/z* 293 $[M+H]^{+}$, 315 $[M+Na]^+$; Anal. Calcd (%) for $C_{16}H_{20}O_5$: C, 65.74; H, 6.90. Found: C, 65.88; H, 6.79.

7-(2,2-Diethoxyethoxy)-2H-chromene-2-one (7d)

White crystals (75%), m.p.68-69°C (DCM); IR (KBr): 3018, 2937, 2843, 1723, 1572 cm^{-1}; ^{1}H-NMR ($CDCl_3$, 300 MHz) δ 1.25 (t, 6H, *J*=7.0 Hz), 3.65 (dq, 2H, J_1=7.1 Hz, J_2=9.4 Hz), 3.78 (dq, 2H, J_1=7.1 Hz, J_2=9.4 Hz), 4.06 (d, 2H, *J*=5.0 Hz), 4.85 (t, 1H, *J*=5.0 Hz), 6.21 (d, 1H, *J*=9.5 Hz), 6.78 (d, 1H, *J*=2.4 Hz), 6.86 (dd, 1H, J_1=2.4 Hz, J_2=8.6 Hz), 7.37 (d, 1H, *J*=8.6 Hz), 7.64 (d, 1H, *J*=9.5 Hz); ^{13}C-NMR ($CDCl_3$, 75 MHz) δ 15.3, 62.8, 68.1, 100.2, 101.9, 107.5, 112.8, 113.3, 128.7, 143.2, 155.8, 160.9, 161.9; MS (ESI): *m/z* 301 $[M+Na]^+$; Anal. Calcd (%) for $C_{15}H_{18}O$: C, 64.74; H, 6.52. Found: C, 64.82; H, 6.45

General Procedure for the Synthesis of (coumarinyloxy)acetaldehydes 8a-d. Synthesis of [(4-methyl-2-oxo-2H-chromen-6-yl)oxy]acetaldehyde (8a).

A solution of acetal **7a** (0.37 g, 1.27 mmol) in 1N HCl (8 ml) was refluxed for 1 h. After cooling the white precipitate was filtered and washed with water to give **8a**, 0.265 g, 94% yield.

[(4-Methyl-2-oxo-2H-chromen-6-yl)oxy]acetaldehyde (8a)

White crystals, m.p.103-104°C (ethyl acetate); IR (KBr): 3027, 2921, 2854, 2741, 1716, 1704, 1615 cm^{-1}; ^{1}H-NMR ($CDCl_3$, 300 MHz) δ 2.40 (s, 3H), 4.62 (s, 2H), 6.31 (s, 1H), 7.06 (d, 1H, *J*=2.7 Hz), 7.11 (dd, 1H, J_1=2.7 Hz, J_2=9.1 Hz), 7.29 (d, 1H, *J*=9.1 Hz), 9.87 (s, 1H); ^{13}C-NMR ($CDCl_3$, 75 MHz) δ 18.6, 73.7, 109.5, 116.1, 118.5, 119.1, 120.9, 148.9, 151.5, 154.3, 160.5, 197.9; MS (ESI): *m/z* 219 $[M+H]^+$; Anal. Calcd (%) for $C_{12}H_{10}O_4$: C, 66.05; H, 4.62. Found: C, 66.12; H, 4.55.

[(2-Oxo-2H-chromen-6-yl)oxy]acetaldehyde (8b)

White crystals (87%), m.p.132-133°C (methanol); IR (KBr): 3026, 2916, 2853, 2748, 1718, 1709, 1596 cm^{-1}; ^{1}H-NMR ($CDCl_3$, 300 MHz) δ 4.62 (s, 2H), 6.45 (d, 1H, *J*=9.4 Hz), 6.91 (d, 1H, *J*=2.7 Hz), 7.15 (dd, 1H, J_1=2.7 Hz, J_2=9.0 Hz), 7.31 (d, 1H, *J*=9.0 Hz), 7.63 (d, 1H, *J*=9.4 Hz), 9.87 (s, 1H); ^{13}C-NMR ($CDCl_3$, 75 MHz) δ 73.4, 111.5, 117.7, 118.4, 119.4, 119.8, 142.8, 149.3, 154.2, 160.6, 198.0; MS (ESI): *m/z* 205 $[M+H]^{+}$, 227 $[M+Na]^+$; Anal. Calcd (%) for $C_{11}H_8O_4$: C, 64.71; H, 3.95. Found: C, 64.65; H, 3.98.

[(4-Methyl-2-oxo-2H-chromen-7-yl)oxy]acetaldehyde (8c)

White crystals (87%), m.p.146-149°C (methanol), m.p. 150-152°C [21].

[(2-Oxo-2H-chromen-7-yl)oxy]acetaldehyde (8d)

White crystals (90%), m.p.123-125°C (methanol), 124-127°C [22].

General Procedure for the Synthesis of (Coumarinyl)Acetaldehyde Oximes. Synthesis of [(4-methyl-2-oxo-2H-chromen-6-yl)oxy]acetaldehyde oxime (9a)

NH_2OH.HCl (0.161 g, 12.34 mmol) and anhydrous CH_3COONa (78 mg, 0.95 mmol) were added to the solution of aldehyde **8a** (0.51 g, 2.34 mmol) in H_2O/EtOH (10:4 ml) and the mixture was heated at 80°C for 1.5 h. After cooling the aqueous solution was eluted with ethyl acetate (8 x 20 ml). The organic layer was dried with anhydrous $MgSO_4$, filtered and concentrated to give **9a** (*E*/*Z*, 3:1), 0.479 g (88% yield).

[(4-Methyl-2-oxo-2H-chromen-6-yl)oxy]acetaldehyde oxime (9a)

White solid, IR (KBr): 3426, 3020, 1710, 1620, 1595 cm^{-1}; ^{1}H-NMR ($CDCl_3$, 300 MHz) (*E/Z, 3:1*) δ 2.40 (s, 9H), 2.42 (s, 3H), 4.70 (d, 6H, *J*=5.5 Hz), 4.94 (d, 2H, *J*=3.5 Hz), 6.29 (s, 3H), 6.32 (s, 1H), 7.04-7.11 (m, 8H), 7.14 (t, 1H, *J*=3.5 Hz), 7.28 (d, 3H, *J*=9.2 Hz), 7.31 (d, 1H, *J*=9.2 Hz), 7.63 (t, 3H, *J*=5.5 Hz); ^{13}C-NMR ($CDCl_3$, 75 MHz) (*E*-) δ 18.6, 65.8, 108.7, 115.9, 118.3, 119.3, 120.7, 146.9, 148.6, 151.8, 154.6, 160.9; MS (ESI): *m/z* 256 $[M+Na]^+$; Anal. Calcd (%) for $C_{12}H_{11}NO_4$: C, 61.80; H, 4.75; N, 6.01. Found: C, 61.96; H, 4.53; N, 5.87.

[(2-oxo-2H-chromen-6-yl)oxy]acetaldehyde oxime (9b)

White solid (84%), IR (KBr): 3387, 3040, 1715, 1620, 1580 cm^{-1}; ^{1}H-NMR ($CDCl_3$, 300 MHz) (*E/Z, 3:1*) δ 4.69 (d, 6H, *J*=5.5 Hz), 4.93 (d, 2H, *J*=3.7 Hz), 6.43 (d, 3H, *J*=9.6 Hz), 6.45 (d, 1H, *J*=9.6 Hz), 6.93 (d, 3H, *J*=2.8 Hz), 6.97 (d,

1H, *J*=2.8 Hz), 7.03 (t, 1H, *J*=3.7 Hz), 7.12 (dd, 3H, J_1=2.8 Hz, J_2=9.0 Hz), 7.16 (dd, 1H, J_1=2.8 Hz, J_2=9.0 Hz), 7.27 (d, 3H, *J*=9.0 Hz), 7.31 (d, 1H, *J*=9.0 Hz), 7.61 (t, 3H, *J*=5.5 Hz), 7.64 (d, 3H, *J*=9.6 Hz), 7.65 (d, 1H, *J*=9.6 Hz); ^{13}C-NMR ($CDCl_3$, 75 MHz) (*E*-) δ 65.8, 111.8, 117.5, 118.2, 119.4, 120.7, 142.9, 146.9, 149.1, 154.6, 160.8; MS (ESI): *m/z* 220 $[M+H]^+$; Anal. Calcd (%) for $C_{11}H_9NO_4$: C, 60.27; H, 4.14; N, 6.39. Found: C, 60.32; H, 4.03; N, 6.31.

[(4-Methyl-2-oxo-2H-chromen-7-yl)oxy]acetaldehyde oxime (9c)

White solid, (87%), IR (KBr): 3372, 3035, 1705, 1610 cm^{-1}; ^{1}H-NMR ($CDCl_3$, 300 MHz) (*E/Z, 3:1*) δ 2.40 (s, 9H), 2.41 (s, 3H), 4.73 (d, 6H, *J*=5.5 Hz), 4.97 (d, 2H, *J*=3.5 Hz), 6.16 (s, 3H), 6.17 (s, 1H), 6.78-6.93 (m, 8H), 7.02 (t, 1H, *J*=3.5 Hz), 7.52 (d, 3H, *J*=8.8 Hz), 7.55 (d, 1H, *J*=8.8 Hz), 7.63 (t, 3H, *J*=5.5 Hz); ^{13}C-NMR ($CDCl_3$, 75 MHz) (*E*-) δ 18.6, 65.6, 102.2, 112.5, 112.7, 114.3, 125.7, 146.6, 148.7, 152.3, 155.3, 161.1; MS (ESI): *m/z* 234 $[M+H]^+$, 256 $[M+Na]^+$; Anal. Calcd (%) for $C_{12}H_{11}NO_4$: C, 61.80; H, 4.75; N, 6.01. Found: C, 61.68; H, 4.64; N, 5.90.

[(2-oxo-2H-chromen-7-yl)oxy]acetaldehyde oxime (9d)

White solid (85%), IR (KBr): 3414, 3020, 1710, 1615 cm^{-1}; ^{1}H-NMR ($CDCl_3$, 300 MHz) (*E/Z, 3:1*) δ 4.72 (d, 6H, *J*=5.7 Hz), 4.96 (d, 2H, *J*=3.7 Hz), 6.16 (d, 3H, *J*=9.5 Hz), 6.19 (d, 1H, *J*=9.5 Hz), 6.74 (d, 3H, *J*=2.3 Hz), 6.78 (d, 1H, *J*=2.3 Hz), 7.00 (t, 1H, *J*=3.7 Hz), 7.31 (dd, 3H, J_1=2.3 Hz, J_2=8.9 Hz), 7.34 (dd, 1H, J_1=2.3 Hz, J_2=8.9 Hz), 7.38 (t, 3H, *J*=5.7 Hz), 7.39 (d, 3H, *J*=8.9 Hz), 7.42 (d, 1H, *J*=8.9 Hz), 7.61 (d, 3H, *J*=9.5 Hz), 7.62 (d, 1H, *J*=9.5 Hz); ^{13}C-NMR ($CDCl_3$, 75 MHz) (*E*-) δ 66.0, 101.6, 112.1, 112.8, 113.3, 128.9, 143.4, 144.8, 148.5, 153.4, 161.0; MS (ESI): *m/z* 242 $[M+Na]^+$; Anal. Calcd (%) for $C_{11}H_9NO_4$: C, 60.27; H, 4.14; N, 6.39. Found: C, 60.15; H, 4.12; N, 6.27.

General Procedure. 1,3-Dipolar Cycloaddition Reactions of (Coumarinyl)Acetaldehyde Oximes with 9-allylpurines. Synthesis of 4-methyl-6-({5-[(6-piperidin-1-yl-9H-purin-9-yl)methyl]-4,5-dihydroisoxazol-3-yl}methoxy)-2H-chromen-2-one (11a).

In the solution of oxime **9a** (37 mg, 0.16 mmol) in dry DMF (5 ml) NCS (32 mg, 0.22 mmol) was added under stirring in portions during 1 h. The resulted mixture was stirred for 30 min. The allylpurine **10a** (39 mg, 0.16 mmol) and Et_3N (0.03 ml, 16 mg, 0.16 mmol) were then added and the mixture was stirred at r.t. for 24 h under N_2 atmosphere. The mixture was filtered, the solid was washed with DCM and the filtrate was evaporated. The residue was chromatographed in a column [hexane/ethyl acetate (2:1)] and gave after the elution of starting purine **10a** (5 mg, 13%) the isoxazoline **11a**, 53 mg, (70% yield).

4-Methyl-6-({5-[(6-piperidin-1-yl-9H-purin-9-yl)methyl]-4,5-dihydroisoxazol-3-yl}methoxy)-2H-chromen-2-one (11a)

White crystals, m.p.148-150°C (ethyl acetate); IR (Nujol): 3080, 1720, 1630, 1565 cm^{-1}; ^{1}H-NMR ($CDCl_3$, 500 MHz) δ 1.69-1.79 (m, 6H), 2.40 (s, 3H), 3.07 (dd, 1H, J_1=6.6 Hz, J_2=17.9 Hz), 3.24 (dd, 1H, J_1=10.9 Hz, J_2=17.9 Hz), 4.23-4.33 (m, 4H), 4.39 (dd, 1H, J_1=5.2 Hz, J_2=14.0 Hz), 4.46 (dd, 1H, J_1=5.4 Hz, J_2=14.0 Hz), 4.76 (s, 2H), 5.03-5.14 (m, 1H), 6.31 (s, 1H), 6.97-7.03 (m, 2H), 7.25 (d, 1H, *J*=8.5 Hz), 7.88 (s, 1H), 8.37 (s, 1H); ^{13}C-NMR ($CDCl_3$, 125 MHz) δ 18.7, 24.0, 26.3, 38.3, 45.6, 49.3, 63.2, 78.6, 109.4, 114.9, 116.0, 118.3, 119.0, 120.8, 124.4, 138.9, 145.6, 149.6, 151.8, 152.3, 154.0, 160.3, 162.1; MS (ESI): *m/z* 475 $[M+H]^+$, 497 $[M+Na]^+$; Anal. Calcd (%) for $C_{25}H_{26}N_6O_4$: C, 63.28; H, 5.52; N, 17.71. Found: C, 63.35; H, 5.47; N, 17.59.

6-({5-[(6-Chloro-9H-purin-9-yl)methyl]-4,5-dihydroisoxazol-3-yl}methoxy)-4-methyl-2H-chromen-2-one (11b)

White crystals (67%), m.p.152-153°C (ethyl acetate); IR (Nujol): 3060, 1710, 1595 cm^{-1}; ^{1}H-NMR ($CDCl_3$, 500 MHz) δ 2.40 (s, 3H), 3.07 (dd, 1H, J_1=7.3 Hz, J_2=17.9 Hz), 3.30 (dd, 1H, J_1=11.1 Hz, J_2=17.9 Hz), 4.37 (dd, 1H, J_1=5.7 Hz, J_2=14.9 Hz), 4.42 (dd, 1H, J_1=3.7 Hz, J_2=14.9 Hz), 4.71 (s, 2H), 5.08-5.17 (m, 1H), 6.31 (s, 1H), 6.90 (d, 1H, *J*=2.9 Hz), 6.95 (dd, 1H, J_1=2.9 Hz, J_2=8.9 Hz), 7.22 (d, 1H, *J*=8.9 Hz), 8.24 (s, 1H), 8.70 (s, 1H); ^{13}C-NMR ($CDCl_3$, 125 MHz) δ 18.6, 37.9, 46.7, 62.9, 78.4, 108.6, 115.9, 118.3, 118.9, 120.6, 131.2, 146.1, 148.6, 151.4, 151.7, 152.0, 153.8, 155.4, 160.6, 161.9; MS (ESI): *m/z* 448/450 $[M+Na]^+$; Anal. Calcd (%) for $C_{20}H_{16}ClN_5O_4$: C, 56.41; H, 3.79; N, 16.45. Found: C, 56.54; H, 3.71; N, 16.56.

6-({5-[(6-Piperidin-1-yl-9H-purin-9-yl)methyl]-4,5-dihydroisoxazol-3-yl}methoxy)-2H-chromen-2-one (11c)

White crystals (70%), m.p.187-189°C (ethyl acetate); IR (Nujol): 3040, 1720, 1610, 1575 cm^{-1}; ^{1}H-NMR ($CDCl_3$,

500 MHz) δ 1.63-1.82 (m, 6H), 3.07 (dd, 1H, J_1=6.9 Hz, J_2=17.6 Hz), 3.23 (dd, 1H, J_1=10.6 Hz, J_2=17.6 Hz), 4.15-4.31 (m, 4H), 4.37 (dd, 1H, J_1=5.5 Hz, J_2=14.2 Hz), 4.41 (dd, 1H, J_1=3.3 Hz, J_2=14.2 Hz), 4.71 (s, 2H), 5.01-5.12 (m, 1H), 6.44 (d, 1H, *J*=9.5 Hz), 6.86 (s, 1H), 7.00 (d, 1H, *J*=9.0 Hz), 7.23 (d, 1H, *J*=9.0 Hz), 7.63 (d, 1H, *J*=9.5 Hz), 7.83 (s, 1H), 8.29 (s, 1H); ^{13}C-NMR ($CDCl_3$, 125 MHz) δ 24.8, 26.2, 37.7, 45.6, 46.6, 63.2, 79.0, 111.4, 117.5, 118.2, 119.3, 119.8, 133.7, 138.9, 142.9, 150.9, 152.4, 153.3, 154.1, 155.5, 160.1, 160.7; MS (ESI): *m/z* 461 $[M+H]^+$; Anal. Calcd (%) for $C_{24}H_{24}N_6O_4$: C, 62.60; H, 5.25; N, 18.25. Found: C, 62.79; H, 5.21; N, 18.12.

4-Methyl-7-({5-[(6-piperidin-1-yl-9H-purin-9-yl)methyl]-4,5-dihydroisoxazol-3-yl}methoxy)-2H-chromen-2-one (11d)

White crystals (72%), m.p.144-145°C (ethyl acetate); IR (Nujol): 3040, 1705, 1570 cm^{-1}; ^{1}H-NMR ($CDCl_3$, 300 MHz) δ 1.70-1.76 (m, 6H), 2.40 (s, 3H), 3.02 (dd, 1H, J_1=6.8 Hz, J_2=18.0 Hz), 3.24 (dd, 1H, J_1=10.8 Hz, J_2=18.0 Hz), 4.21-4.28 (m, 4H), 4.37 (dd, 1H, J_1=5.5 Hz, J_2=14.6 Hz), 4.41 (dd, 1H, J_1=3.6 Hz, J_2=14.6 Hz), 4.71 (d, 1H, *J*=12.8 Hz), 4.77 (d, 1H, *J*=12.8 Hz), 5.02-5.12 (m, 1H), 6.16 (s, 1H), 6.77-6.82 (m, 2H), 7.49 (d, 1H, *J*=9.5 Hz), 7.85 (s, 1H), 8.31 (s, 1H); ^{13}C-NMR ($CDCl_3$, 75 MHz) δ 18.7, 24.8, 26.2, 37.7, 46.0, 46.6, 62.9, 79.0, 102.0, 112.0, 112.7, 114.5, 119.4, 125.8, 138.8, 150.8, 152.3, 152.5, 153.9, 154.9, 155.1, 160.5, 161.0; MS (ESI): *m/z* 475 $[M+H]^+$, 497 $[M+Na]^+$; Anal. Calcd (%) for $C_{25}H_{26}N_6O_4$: C, 63.28; H, 5.52; N, 17.71. Found: C, 63.40; H, 5.46; N, 17.80.

7-({5-[(6-Chloro-9H-purin-9-yl)methyl]-4,5-dihydroisoxazol-3-yl}methoxy)-4-methyl-2H-chromen-2-one (11e)

White crystals (68%), m.p.167-169°C (ethyl acetate); IR (Nujol): 3030, 1715, 1625, 1580 cm^{-1}; ^{1}H-NMR ($CDCl_3$, 300 MHz) δ 2.40 (d, 3H, *J*=1.0 Hz), 3.00 (dd, 1H, J_1=7.0 Hz, J_2=18.1 Hz), 3.31 (dd, 1H, J_1=10.9 Hz, J_2=18.1 Hz), 4.38 (dd, 1H, J_1=5.7 Hz, J_2=14.9 Hz), 4.42 (dd, 1H, J_1=4.0 Hz, J_2=14.9 Hz), 4.75 (s, 2H), 5.07-5.14 (m, 1H), 6.17 (d, 1H, *J*=1.0 Hz), 6.71 (dd, 1H, J_1=2.4 Hz, J_2=8.8 Hz), 6.77 (d, 1H, *J*=2.4 Hz), 7.48 (d, 1H, *J*=8.8 Hz), 8.26 (s, 1H), 8.72 (s, 1H); ^{13}C-NMR ($CDCl_3$, 75 MHz) δ 18.7, 37.9, 46.7, 66.4, 78.3, 102.1, 111.9, 112.9, 114.7, 118.7, 125.9, 131.3, 146.1, 151.4, 152.0, 152.2, 155.0, 155.2, 160.3, 160.8; MS (ESI): *m/z* 448/450 $[M+Na]^+$; Anal. Calcd (%) for $C_{20}H_{16}ClN_5O_4$: C, 56.41; H, 3.79; N, 16.45. Found: C, 56.32; H, 3.84; N, 16.58.

4-Methyl-7-({3-[(6-morpholin-4-yl-9H-purin-9-yl)methyl]-4,5-dihydroisoxazol-5-yl}methoxy)-2H-chromen-2-one (11f)

White crystals (65%), m.p.166-167°C (ethyl acetate); IR (Nujol): 3010, 1700, 1595 cm^{-1}; ^{1}H-NMR ($CDCl_3$, 300 MHz) δ 2.39 (s, 3H), 3.03 (dd, 1H, J_1=6.8 Hz, J_2=17.7 Hz), 3.25 (dd, 1H, J_1=10.8 Hz, J_2=17.7 Hz), 3.76-3.88 (m, 4H), 4.23-4.35 (m, 4H), 4.46 (dd, 1H, J_1=5.6 Hz, J_2=14.6 Hz), 4.51 (dd, 1H, J_1=3.5 Hz, J_2=14.6 Hz), 4.71 (d, 1H, *J*=12.7 Hz), 4.76 (d, 1H, *J*=12.7 Hz), 5.02-5.12 (m, 1H), 6.15 (s, 1H), 6.74-6.87 (m, 2H), 7.50 (d, 1H, *J*=8.1 Hz), 7.86 (s, 1H), 8.33 (s, 1H); ^{13}C-NMR ($CDCl_3$, 75 MHz) δ 18.6, 37.7, 45.9, 46.2, 62.9, 67.0, 78.9, 102.1, 112.0, 112.7, 114.5, 119.6, 125.8, 139.6, 150.8, 152.0, 152.2, 153.8, 154.9, 155.0, 160.4, 160.9; MS (ESI): *m/z* 477 $[M+H]^+$; Anal. Calcd (%) for $C_{24}H_{24}N_6O_5$: C, 60.50; H, 5.08; N, 17.64. Found: C, 60.58; H, 5.14; N, 17.55.

7-({5-[(6-Piperidin-1-yl-9H-purin-9-yl)methyl]-4,5-dihydroisoxazol-3-yl}methoxy)-2H-chromen-2-one (11g)

White crystals (62%), m.p.184-185°C (ethyl acetate); IR (Nujol): 3010, 1700, 1615, 1565 cm^{-1}; ^{1}H-NMR ($CDCl_3$, 300 MHz) δ 1.60-1.76 (m, 6H), 3.03 (dd, 1H, J_1=7.0 Hz, J_2=17.8 Hz), 3.24 (dd, 1H, J_1=10.6 Hz, J_2=17.8 Hz), 4.18-4.29 (m, 4H), 4.36 (dd, 1H, J_1=5.4 Hz, J_2=14.8 Hz), 4.43 (dd, 1H, J_1=3.8 Hz, J_2=14.8 Hz), 4.70 (d, 1H, *J*=12.5 Hz), 4.76 (d, 1H, *J*=12.5 Hz), 5.02-5.11 (m, 1H), 6.27 (d, 1H, *J*=9.5 Hz), 6.73-6.80 (m, 2H), 7.36 (d, 1H, *J*=8.9 Hz), 7.62 (d, 1H, *J*=9.5 Hz), 7.84 (s, 1H), 8.31 (s, 1H); ^{13}C-NMR ($CDCl_3$, 75 MHz) δ 22.7, 26.2, 37.7, 46.2, 46.8, 63.0, 79.1, 102.1, 112.4, 113.5, 114.0, 119.3, 129.0, 139.0, 143.7, 149.9, 152.0, 152.5, 153.1, 154.8, 155.0, 160.7, 161.4; MS (ESI): *m/z* 461 $[M+H]^+$, 483 $[M+Na]^+$; Anal. Calcd (%) for $C_{24}H_{24}N_6O_4$: C, 62.60; H, 5.25; N, 18.25. Found: C, 62.71; H, 5.20; N, 18.16.

General Procedure. 1,3-Dipolar Cycloaddition Reactions of (purin-9-yl)Acetaldehyde Oximes with Propargyloxycoumarins. Synthesis of 4-methyl-6-({3-[(6-piperidin-1-yl-9H-purin-9-yl)methyl]isoxazol-5-yl}methoxy)-2H-chromen-2-one (13a)

TFA (4 μl, 5.7 mg, 0.05 mmol) was added to the solution of propargyloxycoumarin **12a** (56 mg, 0.26 mmol) and PIDA (84 mg, 0.26 mmol) in methanol (3 ml). Then, in the resulted mixture, a solution of oxime **2a** (62 mg, 0.24

mmol) in methanol (2 ml) was transferred dropwise during 1.5 h and the mixture was stirred at r.t. for 4 h. The solvent was evaporated and the solid residue was separated by column chromatography [hexane/ethyl acetate (2:1)] followed by PTLC (ethyl acetate) to give the aldehyde **1a** (5 mg, 9%) and the isoxazole **13a** (73 mg, 64%), while the furoxan **5a** (20 mg, 16%) [18] eluted next.

When the reaction of **2a** and **12a** was performed according to the general procedure *5.1.3.*, the isoxazole **13a** (28%), the furoxan **5a** (25%) and the starting oxime **2a** (45%) were isolated from the reaction mixture.

4-Methyl-6-({3-[(6-piperidin-1-yl-9H-purin-9-yl)methyl]isoxazol-5-yl}methoxy)-2H-chromen-2-one (13a)

White crystals, m.p.151-152°C (DCM); IR (Nujol): 3030, 1710, 1620, 1570 cm^{-1}; ^{1}H-NMR ($CDCl_3$, 500 MHz) δ 1.68-1.82 (m, 6H), 2.40 (s, 3H), 4.21-4.38 (m, 4H), 5.16 (s, 2H), 5.48 (s, 2H), 6.32 (s, 1H), 6.44 (s, 1H), 7.06-7.17 (m, 2H), 7.29 (d, 1H, *J*=8.7 Hz), 7.83 (s, 1H), 8.40 (s, 1H); ^{13}C-NMR ($CDCl_3$, 125 MHz) δ 18.6, 26.2, 29.7, 38.9, 47.2, 62.1, 103.4, 109.8, 116.0, 118.3, 119.3, 119.6, 120.8, 138.1, 148.9, 150.1, 151.5, 152.2, 154.0, 159.6, 160.5, 160.7, 168.8; MS (ESI): *m/z* 473 $[M+H]^+$, 495 $[M+Na]^+$; Anal. Calcd (%) for $C_{25}H_{24}N_6O_4$: C, 63.55; H, 5.12; N, 17.79. Found: C, 63.62; H, 5.17; N, 17.63.

4-Methyl-6-({3-[(6-morpholin-4-yl-9H-purin-9-yl)methyl]isoxazol-5-yl}methoxy)-2H-chromen-2-one (13b)

White crystals, m.p.166-169°C (DCM); IR (Nujol): 3030, 1725, 1605, 1580 cm^{-1}; ^{1}H-NMR ($CDCl_3$, 300 MHz) δ 2.38 (s, 3H), 3.74-3.89 (m, 4H), 4.22-4.40 (m, 4H), 5.15 (s, 2H), 5.47 (s, 2H), 6.29 (s, 1H), 6.41 (s, 1H), 7.03-7.16 (m, 2H), 7.26 (d, 1H, *J*=9.3 Hz), 7.83 (s, 1H), 8.39 (s, 1H); ^{13}C-NMR ($CDCl_3$, 75 MHz) δ 18.6, 38.6, 45.9, 62.0, 67.0, 103.2, 109.7, 115.9, 118.3, 119.2, 120.7, 138.3, 148.8, 150.7, 151.5, 152.4, 153.9, 154.0, 158.9, 159.6, 160.5, 168.7; MS (ESI): *m/z* 475 $[M+H]^+$, 497 $[M+Na]^+$; Anal. Calcd (%) for $C_{24}H_{22}N_6O_5$: C, 60.75; H, 4.67; N, 17.71. Found: C, 60.70; H, 4.54; N, 17.51.

3,4-bis-[(6-Morpholin-4-yl-9H-purin-9-yl)methyl]-1,2,5-oxadiazole-2-oxide (5b)

White crystals (18%), m.p.66-67°C (DCM); IR (Nujol): 3030, 1620, 1590, 1530 cm^{-1}; ^{1}H-NMR ($CDCl_3$, 300 MHz) δ 3.78-3.91 (m, 8H), 4.22-4.43 (m, 8H), 5.53 (s, 2H), 5.96 (s, 2H), 7.93 (s, 1H), 8.02 (s, 1H), 8.32 (s, 1H), 8.34 (s, 1H); ^{13}C-NMR ($CDCl_3$, 75 MHz) δ 36.2, 38.9, 45.8, 66.8, 111.6, 119.5, 119.7, 138.3, 138.4, 150.0, 150.2, 151.3, 151.7, 152.3, 152.6, 153.1; MS (ESI): *m/z* 521 $[M+H]^+$; Anal. Calcd (%) for $C_{22}H_{24}N_{12}O_4$: C, 50.77; H, 4.65; N, 32.29. Found: C, 50.72; H, 4.57; N%,32.18.

4-Methyl-7-({3-[(6-piperidin-1-yl-9H-purin-9-yl)methyl]isoxazol-5-yl}methoxy)-2H-chromen-2-one (13c)

White crystals (57%), m.p.165-167°C (DCM); IR (Nujol): 3035, 1710, 1620, 1560 cm^{-1}; ^{1}H-NMR ($CDCl_3$, 300 MHz) δ 1.65-1.79 (m, 6H), 2.38 (s, 3H), 4.19-4.31 (m, 4H), 5.15 (s, 2H), 5.44 (s, 2H), 6.16 (s, 1H), 6.40 (s, 1H), 6.82-6.90 (m, 2H), 7.50 (d, 1H, *J*=8.5 Hz), 7.79 (s, 1H), 8.36 (s, 1H); ^{13}C-NMR ($CDCl_3$, 75 MHz) δ 18.8, 26.2, 29.7, 38.9, 47.7, 61.2, 101.9, 103.5, 112.5, 112.8, 114.7, 119.3, 126.0, 128.9, 129.8, 138.5, 151.4, 152.4, 155.1, 159.3, 160.3, 161.0, 168.3; MS (ESI): *m/z* 473 $[M+H]^+$; Anal. Calcd (%) for $C_{25}H_{24}N_6O_4$: C, 63.55; H, 5.12; N, 17.79. Found: C, 63.62; H, 5.04; N, 17.68.

4-Methyl-7-({3-[(6-morpholin-4-yl-9H-purin-9-yl)methyl]isoxazol-5-yl}methoxy)-2H-chromen-2-one (13d)

White crystals (54%), m.p.179-180°C (DCM); IR (Nujol): 3050, 1720, 1605, 1575 cm^{-1}; ^{1}H-NMR ($CDCl_3$, 300 MHz) δ 2.40 (s, 3H), 3.81-3.88 (m, 4H), 4.28-4.42 (m, 4H), 5.16 (s, 2H), 5.50 (s, 2H), 6.17 (s, 1H), 6.45 (s, 1H), 6.84 (d, 1H, *J*=2.5 Hz), 6.88 (dd, 1H, J_1=2.5 Hz, J_2=8.7 Hz), 7.51 (d, 1H, *J*=8.7 Hz), 7.85 (s, 1H), 8.42 (s, 1H); ^{13}C-NMR ($CDCl_3$, 75 MHz) δ 18.4, 38.6, 45.9, 61.2, 66.9, 102.0, 103.3, 112.5, 114.6, 119.7, 125.9, 138.7, 150.4, 151.8, 152.7, 153.1, 153.6, 155.0, 159.5, 160.5, 161.3, 168.2; MS (ESI): *m/z* 475 $[M+H]^+$; Anal. Calcd (%) for $C_{24}H_{22}N_6O_5$: C, 60.75; H, 4.67; N, 17.71. Found: C, 60.83; H, 4.62; N. 17.65.

7-({3-[(6-Piperidin-1-yl-9H-purin-9-yl)methyl]isoxazol-5-yl}methoxy)-2H-chromen-2-one (13e)

White crystals (56%), m.p.154-156°C (DCM); IR (Nujol): 3020, 1710, 1615, 1565 cm^{-1}; ^{1}H-NMR ($CDCl_3$, 300 MHz) δ 1.67-1.81 (m, 6H), 4.17-4.33 (m, 4H), 5.15 (s, 2H), 5.45 (s, 2H), 6.27 (d, 1H, *J*=9.5 Hz), 6.41 (s, 1H), 6.81-6.89 (m, 2H), 7.38 (d, 1H, *J*=8.3 Hz), 7.61 (d, 1H, *J*=9.5 Hz), 7.79 (s, 1H), 8.36 (s, 1H); ^{13}C-NMR ($CDCl_3$, 75 MHz) δ 24.7,

26.2, 38.7, 46.9, 61.4, 102.1, 103.5, 112.7, 113.6, 114.1, 119.6, 129.1, 138.6, 143.0, 150.4, 152.1, 153.3, 155.8, 159.6, 160.6, 161.2, 168.1; MS (ESI): *m/z* 459 $[M+H]^+$; Anal. Calcd (%) for $C_{24}H_{22}N_6O_4$: C, 62.87; H, 4.84; N, 18.33. Found: C, 62.92; H, 4.89; N, 18.22.

7-({3-[(6-Morpholin-4-yl-9H-purin-9-yl)methyl]isoxazol-5-yl}methoxy)-2H-chromen-2-one (13f)

White crystals (53%), m.p.174-175°C (DCM); IR (Nujol): 3040, 1705, 1610, 1540 cm^{-1}; ^{1}H-NMR ($CDCl_3$, 300 MHz) δ 3.80-3.89 (m, 4H), 4.34-4.42 (m, 4H), 5.17 (s, 2H), 5.53 (s, 2H), 6.28 (d, 1H, *J*=9.5 Hz), 6.47 (s, 1H), 6.82-6.90 (m, 2H), 7.40 (d, 1H, *J*=8.5 Hz), 7.63 (d, 1H, *J*=9.5 Hz), 7.89 (s, 1H), 8.43 (s, 1H); ^{13}C-NMR ($CDCl_3$, 75 MHz) δ 38.7, 45.8, 61.3, 67.0, 101.9, 103.4, 112.7, 113.5, 114.1, 119.1, 129.1, 138.3, 143.2, 150.6, 152.3, 153.2, 155.7, 159.5, 160.5, 160.8, 168.1; MS (ESI): *m/z* 461 $[M+H]^+$; Anal. Calcd (%) for $C_{23}H_{20}N_6O_5$: C, 60.00; H, 4.38; N, 18.25. Found: C, 60.12; H, 4.19; N, 18.16.

7-({3-[(6-Pyrrolidin-1-yl-9H-purin-9-yl)methyl]isoxazol-5-yl}methoxy)-2H-chromen-2-one (13g)

White crystals (53%), m.p.176-178°C (DCM); IR (Nujol): 3010, 1725, 1590, 1545 cm^{-1}; ^{1}H-NMR ($CDCl_3$, 300 MHz) δ 1.98-2.07 (m, 4H), 3.73-3.86 (m, 2H), 4.12-4.25 (m, 2H), 5.16 (s, 2H), 5.46 (s, 2H), 6.29 (d, 1H, *J*=9.5 Hz), 6.42 (s, 1H), 6.81-6.90 (m, 2H), 7.40 (d, 1H, *J*=8.4 Hz), 7.64 (d, 1H, *J*=9.5 Hz), 7.82 (s, 1H), 8.40 (s, 1H); ^{13}C-NMR ($CDCl_3$, 75 MHz) δ 30.9, 38.6, 47.9, 61.4, 102.2, 103.4, 112.7, 113.7, 114.2, 120.1, 129.1, 138.6, 143.0, 150.1, 152.4, 152.7, 155.8, 159.8, 160.6, 161.9, 168.1; MS (ESI): *m/z* 445 $[M+H]^+$, 467 $[M+Na]^+$; Anal. Calcd (%) for $C_{23}H_{20}N_6O_4$: C, 62.16; H, 4.54; N, 18.91. Found: C, 62.29; H, 4.48; N, 18.79.

3,4-bis-[(6-Pyrrolidin-1-yl-9H-purin-9-yl)methyl]-1,2,5-oxadiazole-2-oxide (5c)

White crystals (17%), m.p.71-72°C (DCM); IR (Nujol): 3030, 1610, 1595, 1540 cm^{-1}; ^{1}H-NMR ($CDCl_3$, 300 MHz) δ 1.94-2.12 (m, 4H), 3.93-4.26 (m, 4H), 5.51 (s, 2H), 5.98 (s, 2H), 7.96 (s, 1H), 8.04 (s, 1H), 8.33 (s, 1H), 8.36 (s, 1H); ^{13}C-NMR ($CDCl_3$, 75 MHz) δ 30.8, 36.2, 38.9, 44.2, 45.2, 111.9, 119.9, 120.4, 137.9, 138.2, 150.1, 150.5, 151.1, 151.9, 152.6, 152.8, 153.2; MS (ESI): *m/z* 489 $[M+H]^+$; Anal. Calcd (%) for $C_{22}H_{24}N_{12}O_2$: C, 54.09; H, 4.95; N, 34.47. Found: C, 54.03; H, 4.97; N, 34.38.

4-({3-[(6-Piperidin-1-yl-9H-purin-9-yl)methyl]isoxazol-5-yl}methoxy)-2H-chromen-2-one (13h)

White crystals (60%), m.p.161-163°C (DCM); IR (KBr): 3026, 2912, 1723, 1608 cm^{-1}; ^{1}H-NMR ($CDCl_3$, 300 MHz) δ 1.64-1.81 (m, 6H), 4.18-4.35 (m, 4H), 5.24 (s, 2H), 5.49 (s, 2H), 5.74 (s, 1H), 6.55 (s, 1H), 7.21-7.35 (m, 2H), 7.55 (t, 1H, *J*=8.4 Hz), 7.77 (d, 1H, *J*=7.9 Hz), 7.83 (s, 1H), 8.38 (s, 1H); ^{13}C-NMR ($CDCl_3$, 75 MHz) δ 24.5, 26.2, 39.0, 47.6, 61.4, 91.6, 104.3, 115.2 116.9, 119.6, 123.1, 124.2, 132.9, 138.4, 150.8, 152.4, 153.5, 154.1, 159.6, 162.0, 164.5, 166.5; MS (ESI): *m/z* 459 $[M+H]^+$, 481 $[M+Na]^+$; Anal. Calcd (%) for $C_{24}H_{22}N_6O_4$: C, 62.87; H, 4.84; N, 18.33. Found: C, 62.96; H, 4.76; N, 18.24.

4-({3-[(6-Morpholin-4-yl-9H-purin-9-yl)methyl]isoxazol-5-yl}methoxy)-2H-chromen-2-one (13i)

White crystals (58%), m.p.159-161°C (DCM); IR (KBr): 3016, 2928, 1706, 1614, 1587 cm^{-1}; ^{1}H-NMR ($CDCl_3$, 300 MHz) δ 3.78-3.93 (m, 4H), 4.23-4.36 (m, 4H), 5.28 (s, 2H), 5.50 (s, 2H), 5.78 (s, 1H), 6.57 (s, 1H), 7.17-7.32 (m, 2H), 7.57 (t, 1H, *J*=8.7 Hz), 7.78 (d, 1H, *J*=7.4 Hz), 7.89 (s, 1H), 8.37 (s, 1H); ^{13}C-NMR ($CDCl_3$, 75 MHz) δ 38.7, 46.0, 61.3, 67.0, 91.5, 104.5, 115.1, 116.8, 116.9, 123.0, 124.2, 132.9, 138.4, 150.5, 152.1, 153.5, 153.7, 159.7, 162.0, 164.5, 166.4; MS (ESI): *m/z* 461 $[M+H]^+$; Anal. Calcd (%) for $C_{23}H_{20}N_6O_5$: C, 60.00; H, 4.38; N, 18.25. Found: C, 60.07; H, 4.31; N, 18.20.

General Procedure for the Synthesis of 9-propargylpurines 14a-c. Synthesis of 6-piperidin-1-yl-9-prop-2-yn-1-yl-9H-purine (14a)

Anhydrous K_2CO_3 (0.297 g, 2.1 mmol) was added to the solution of 6-piperidin-1-yl-9H-purine (.43 g, 2.1 mmol) in DMF (15 ml) followed by propargyl bromide (0.16 ml, 0.25 g, 3.32 mmol). The mixture was heated at 90°C for 24 h and filtered, while hot. The solid K_2CO_3 was washed with DCM and the filtrates were evaporated. The residue was chromatographed in a column [hexane/ethyl acetate (1:1)] to give compound **14a** (0.641 g, 80%)

6-Piperidin-1-yl-9-prop-2-yn-1-yl-9H-purine (14a)

White crystals, m.p. 99-100°C (DCM); IR (KBr): 3024, 2963, 2878, 2143, 1584, 1506 cm^{-1}; ^{1}H-NMR ($CDCl_3$, 500 MHz) δ 1.58-1.84 (m, 6H), 2.48 (t, 1H, *J*=2.5 Hz), 4.12-4.34 (m, 4H), 4.94 (d, 2H, *J*=2.5 Hz), 7.91 (s, 1H), 8.34 (s, 1H); ^{13}C-NMR ($CDCl_3$, 125 MHz) δ 24.9, 26.1, 41.5, 46.5, 74.6, 78.4, 119.8, 137.0, 150.5, 152.7, 154.0; MS (ESI): *m/z* 242 $[M+H]^+$; Anal. Calcd (%) for $C_{13}H_{15}N_5$: C, 64.71; H, 6.27; N, 29.02. Found: C, 64.93; H, 6.42; N, 28.94.

6-Morpholin-4-yl-9-prop-2-yn-1-yl-9H-purine (14b)

White crystals (78%), m.p. 160-161°C (DCM); IR (KBr): 3035, 2946, 2862, 2127, 1589, 1534 cm^{-1}; ^{1}H-NMR ($CDCl_3$, 300 MHz) δ 2.49 (t, 1H, *J*=2.5 Hz), 3.77-3.88 (m, 4H), 4.25-4.36 (m, 4H), 4.96 (d, 2H, *J*=2.5 Hz), 7.93 (s, 1H), 8.37 (s, 1H); ^{13}C-NMR ($CDCl_3$, 75 MHz) δ 41.2, 45.8, 67.0, 74.8, 78.2, 119.9, 137.6, 150.6, 152.5, 153.9; MS (ESI): *m/z* 244 $[M+H]^+$; Anal. Calcd (%) for $C_{12}H_{13}N_5O$: C, 59.25; H, 5.39; N, 28.79. Found: C, 59.32; H, 5.23; N, 28.57.

6-Pyrrolidin-1-yl-9-prop-2-yn-1-yl-9H-purine (14c)

White crystals, m.p. 139-141°C (DCM); IR (KBr): 3052, 2946, 2867, 2123, 1608, 1534 cm^{-1}; ^{1}H-NMR ($CDCl_3$, 300 MHz) δ 1.95-2.09 (m, 4H), 2.56 (t, 1H, *J*=2.5 Hz), 3.98-4.12 (m, 4H), 4.96 (d, 2H, *J*=2.5 Hz), 7.92 (s, 1H), 8.34 (s, 1H); ^{13}C-NMR ($CDCl_3$, 75 MHz) δ 31.1, 40.1, 48.0, 48.6, 74.4, 78.3, 119.9, 137.6, 149.6, 152.7, 152.9; MS (ESI): *m/z* 228 $[M+H]^+$; Anal. Calcd (%) for $C_{12}H_{13}N_5$: C, 63.42; H, 5.77; N, 30.82. Found: C, 63.51; H, 5.68; N, 30.94.

General Procedure. 1,3-Dipolar Cycloaddition Reactions of [(2-oxo-2H-chromen-7-yl)oxy]acetaldehyde oxime (9d) with propargylpurines. Synthesis of 7-({5-[(6-piperidin-1-yl-9H-purin-9-yl)methyl]isoxazol-3-yl}methoxy)-2H-chromen-2-one (15a).

TFA (4 µl, 5.7 mg, 0.05 mmol) was added to the solution of propargylpurine **14a** (63 mg, 0.26 mmol) and PIDA (84 mg, 0.26 mmol) in methanol (3 ml). Then, in the resulted mixture, a solution of oxime **9d** (53 mg, 0.24 mmol) in methanol (2 ml) was transferred dropwise during 1.5 h and the mixture was stirred at r.t. for 4 h. The solvent was evaporated and the solid residue was separated by column chromatography [hexane/ethyl acetate (2:1)] followed by PTLC (ethyl acetate) to give the aldehyde **8d** (5 mg, 11%) and the isoxazole **15a** (62 mg, 56%), while the furoxan **16** (20 mg, 19%) eluted next.

7-({5-[(6-Piperidin-1-yl-9H-purin-9-yl)methyl]isoxazol-3-yl}methoxy)-2H-chromen-2-one (15a)

White crystals, m.p. 140-142°C (DCM); IR (Nujol): 3040, 1725, 1595 cm^{-1}; ^{1}H-NMR ($CDCl_3$, 500 MHz) δ 1.65-1.77 (m, 6H), 4.18-4.35 (m, 4H), 5.16 (s, 2H), 5.52 (s, 2H), 6.25 (d, 1H, *J*=9.5 Hz), 6.41 (s, 1H), 6.80-6.93 (m, 2H), 7.36 (d, 1H, *J*=9.2 Hz), 7.60 (d, 1H, *J*=9.5 Hz), 7.85 (s, 1H), 8.37 (s, 1H); ^{13}C-NMR ($CDCl_3$, 125 MHz) δ 24.7, 26.2, 38.7, 47.0, 62.0, 102.3, 103.1, 112.7, 113.5, 114.0, 119.6, 129.1, 137.8, 143.1, 149.7, 151.7, 153.4, 155.9, 160.4, 160.7, 161.0, 166.8; MS (ESI): *m/z* 459 $[M+H]^+$, 497 $[M+K]^+$; Anal. Calcd (%) for $C_{24}H_{22}N_6O_4$: C, 62.87; H, 4.84; N, 18.33. Found: C, 62.93; H, 4.78; N, 18.17.

7,7'-[(2-oxido-1,2,5-oxadiazole-3,4-diyl)bis(methyleneoxy)]bis(2H-chromen-2-one) (16)

White crystals, m.p. 168-170°C (DCM); IR (Nujol): 3020, 1710, 1605, 1565 cm^{-1}; ^{1}H-NMR ($CDCl_3$, 500 MHz) δ 5.16 (s, 2H), 5.29 (s, 2H), 6.27 (d, 2H, *J*=9.5 Hz), 6.76-6.93 (m, 4H), 7.39 (d, 2H, *J*=7.7 Hz), 7.61 (d, 2H, *J*=9.5 Hz); ^{13}C-NMR ($CDCl_3$, 125 MHz) δ 59.0, 61.6, 102.4, 102.5, 111.5, 112.1, 112.3, 114.0, 114.1, 114.7, 114.9, 129.3, 129.4, 142.4, 142.9, 153.8, 155.8, 155.9, 160.0, 160.2, 160.4, 160.7; MS (ESI): *m/z* 435 $[M+H]^+$; Anal. Calcd (%) for $C_{24}H_{14}N_2O_8$: C, 60.83; H, 3.25; N, 6.46. Found: C, 60.95; H, 3.17; N, 6.41.

7-({5-[(6-Morpholin-4-yl-9H-purin-9-yl)methyl]isoxazol-3-yl}methoxy)-2H-chromen-2-one (15b)

White crystals (59%), m.p.185-187°C (DCM); IR (Nujol): 3010, 1705, 1590 cm^{-1}; ^{1}H-NMR ($CDCl_3$, 500 MHz) δ 3.79-3.87 (m, 4H), 4.26-4.38 (m, 4H), 5.16 (s, 2H), 5.52 (s, 2H), 6.27 (d, 1H, *J*=9.5 Hz), 6.41 (s, 1H), 6.84-6.93 (m, 2H), 7.37 (d, 1H, *J*=9.2 Hz), 7.61 (d, 1H, *J*=9.5 Hz), 7.87 (s, 1H), 8.38 (s, 1H); ^{13}C-NMR ($CDCl_3$, 125 MHz) δ 39.1, 47.0, 62.0, 66.9, 102.2, 103.8, 112.8, 113.6, 114.1, 119.0, 129.1, 139.1, 143.0, 149.6, 152.2, 153.6, 155.6, 159.9, 160.5, 160.9, 165.9; MS (ESI): *m/z* 461 $[M+H]^+$; Anal. Calcd (%) for $C_{23}H_{20}N_6O_5$: C, 60.00; H. 4.38; N, 18.25. Found: C, 60.14; H, 4.42; N, 18.14.

7-({5-[(6-Pyrrolidin-1-yl-9H-purin-9-yl)methyl]isoxazol-3-yl}methoxy)-2H-chromen-2-one (15c)

White crystals (53%),, m.p.172-173°C (DCM); IR (Nujol): 3050, 1710, 1620, 1575 cm^{-1}; ^{1}H-NMR ($CDCl_3$, 300 MHz) δ 1.96-2.03 (m, 4H), 3.76-4.19 (m, 4H), 5.16 (s, 2H), 5.51 (s, 2H), 6.25 (d, 1H, *J*=9.5 Hz), 6.38 (s, 1H), 6.78-6.92 (m, 2H), 7.36 (d, 1H, *J*=9.0 Hz), 7.61 (d, 1H, *J*=9.5 Hz), 7.86 (s, 1H), 8.38 (s, 1H); ^{13}C-NMR ($CDCl_3$, 75 MHz) δ 30.5, 48.2, 49.6, 62.0, 102.3, 103.0, 112.7, 113.5, 113.9, 119.9, 129.0, 138.7, 143.1, 149.6, 152.0, 152.1, 155.8, 160.3, 160.7, 160.9, 166.9; MS (ESI): *m/z* 445 $[M+H]^+$, 483 $[M+K]^+$; Anal. Calcd (%) for $C_{23}H_{20}N_6O_4$: C, 62.16; H, 4.54; N, 18.91. Found: C, 62.22; H, 4.46; N, 18.83.

General Procedure for the Synthesis of 9-vinylpurines 17a-c. Synthesis of 6-piperidin-1-yl-9-vinyl-9H-purine (17a).

Anhydrous K_2CO_3 (0.894 g, 6.5 mmol) and 1,2-dibromoethane (0.47 ml, 0.706 g, 6.98 mmol) were added to the solution of *6-piperidin-1-yl-9H-purine* (0.66 g, 3.25 mmol) in dry DMF (10 ml). The mixture was heated at 90°C under N_2 atmosphere for 24 h and filtered while hot. The precipitate was washed with DCM and the filtrate was evaporated. The solid residue was separated by column chromatography [hexane/ethyl acetate (1:1)] to give vinylpurine **17a** (89 mg, 12% yield) followed by 9-(2-bromoethyl)-6-piperidin-1-yl-9*H*-purine (0.534 g, 72% yield).

6-Piperidin-1-yl-9-vinyl-9H-purine (17a)

White crystals, m.p. 56-58°C (DCM); IR (KBr): 3028, 2927, 2834, 1627, 1556 cm^{-1}; ^{1}H-NMR ($CDCl_3$, 300 MHz) δ 1.64-1.81 (m, 6H), 4.17-4.31 (m, 4H), 5.10 (dd, 1H, J_1=1.3 Hz, J_2=9.1 Hz), 5.75 (dd, 1H, J_1=1.3 Hz, J_2=16.1 Hz), 7.25 (dd, 1H, J_1=9.1 Hz, J_2=16.1 Hz), 7.94 (s, 1H), 8.35 (s, 1H); ^{13}C-NMR ($CDCl_3$, 75 MHz) δ 24.8, 26.2, 46.6, 103.4, 120.2, 126.7, 134.9, 150.2, 153.0, 153.9; MS (ESI): *m/z* 230 $[M+H]^+$; Anal. Calcd (%) for $C_{12}H_{15}N_5$: C, 62.86; H, 6.59; N, 30.54. Found: C, 63.06; H, 6.45; N, 30.37.

6-Morpholin-4-yl-9-vinyl-9H-purine (17b)

White crystals (10%), m.p. 60-62°C (DCM); IR (KBr): 3056, 2947, 2846, 1583, 1504 cm^{-1}; ^{1}H-NMR ($CDCl_3$, 300 MHz) δ 3.78-3.91 (m, 4H), 4.26-4.35 (m, 4H), 5.15 (d, 1H, *J*=9.1 Hz), 5.79 (d, 1H, *J*=16.1 Hz), 7.26 (dd, 1H, J_1=9.1 Hz, J_2=16.1 Hz), 7.96 (s, 1H), 8.39 (s, 1H); ^{13}C-NMR ($CDCl_3$, 75 MHz) δ 46.0, 65.6, 103.9, 120.0, 126.7, 135.7, 149.9, 152.6, 155.2; MS (ESI): *m/z* 232 $[M+H]^+$; Anal. Calcd (%) for $C_{11}H_{13}N_5O$: C, 57.13; H, 5.67; N, 30.28. Found: C, 57.24; H, 5.52; N, 30.12.

6-Pyrrolidin-1-yl-9-vinyl-9H-purine (17c)

White crystals, m.p. 64-66°C (DCM); IR (KBr): 3012, 2923, 1615, 1536 cm^{-1}; ^{1}H-NMR ($CDCl_3$, 300 MHz) δ 1.86-2.08 (m, 4H), 4.89-4.38 (m, 4H), 5.12 (dd, 1H, J_1=1.1 Hz, J_2=9.0 Hz), 5.72 (dd, 1H, J_1=1.1 Hz, J_2=15.9 Hz), 7.22 (dd, 1H, J_1=9.0 Hz, J_2=15.9 Hz), 7.87 (s, 1H), 8.36 (s, 1H); ^{13}C-NMR ($CDCl_3$, 75 MHz) δ 32.8, 44.3, 102.8, 119.8, 126.2, 137.4, 150.1, 152.8, 153.1; MS (ESI): *m/z* 216 $[M+H]^+$; Anal. Calcd (%) for $C_{11}H_{13}N_5$: C, 61.38; H, 6.09; N, 32.54. Found: C, 61.46; H, 6.12; N, 32.47.

General Procedure. 1,3-Dipolar Cycloaddition Reactions of [(2-oxo-2H-chromen-7-yl)oxy]acetaldehyde oxime (9d) with vinylpurines. Synthesis of 7-{[5-(6-piperidin-1-yl-9H-purin-9-yl)-4,5-dihydroisoxazol-3-yl]methoxy}-2H-chromen-2-one (18a)

TFA (4 μl, 5.7 mg, 0.05 mmol) was added to the solution of vinylpurine **17a** (60 mg, 0.26 mmol) and PIDA (84 mg, 0.26 mmol) in methanol (3 ml). Then, in the resulted mixture, a solution of oxime **9d** (53 mg, 0.24 mmol) in methanol (2 ml) was transferred dropwise during 1.5 h and the mixture was stirred at r.t. for 4 h. The solvent was evaporated and the solid residue was separated by column chromatography [hexane/ethyl acetate (2:1)] followed by PTLC (ethyl acetate) to give the aldehyde **8d** (7 mg, 15%) and the isoxazoline **18a** (71 mg, 66%).

7-{[5-(6-Piperidin-1-yl-9H-purin-9-yl)-4,5-dihydroisoxazol-3-yl]methoxy}-2H-chromen-2-one (18a)

White crystals, m.p. 197-199°C (ethyl acetate); IR (Nujol): 3040, 1710, 1585 cm^{-1}; ^{1}H-NMR ($CDCl_3$, 500 MHz) δ 1.67-1.81 (m, 6H), 3.68-3.75 (m, 2H), 4.22-4.34 (m, 4H), 5.06 (d, 1H, *J*=12.8 Hz), 5.12 (d, 1H, *J*=12.8 Hz), 6.29 (d, 1H, *J*=9.6 Hz), 6.78-6.85 (m, 1H), 6.89-6.95 (m, 2H), 7.41 (d, 1H, *J*=9.1 Hz), 7.63 (d, 1H, *J*=9.6 Hz), 7.77 (s, 1H), 8.29 (s,

1H); ^{13}C-NMR ($CDCl_3$, 125 MHz) δ 24.7, 26.2, 41.7, 46.9, 63.1, 84.4, 102.5, 112.4, 113.7, 114.3, 120.3, 129.2, 136.0, 143.0, 149.7, 152.0, 153.4, 155.8, 155.9, 160.6, 160.7; MS (ESI): *m/z* 447 $[M+H]^+$; Anal. Calcd (%) for $C_{23}H_{22}N_6O_4$: C, 61.87; H, 4.97; N, 18.82. Found: C, 61.95; H, 4.93; N, 18.72.

7-{[5-(6-Morpholin-4-yl-9H-purin-9-yl)-4,5-dihydroisoxazol-3-yl]methoxy}-2H-chromen-2-one (18b)

White crystals (62%), m.p. 205-206°C (ethyl acetate); IR (Nujol): 3010, 1720, 1590 cm^{-1}; ^{1}H-NMR ($CDCl_3$, 300 MHz) δ 3.70-3.76 (m, 2H), 3.78-3.89 (m, 4H), 4.29-4.38 (m, 4H), 5.06 (d, 1H, *J*=12.3 Hz), 5.12 (d, 1H, *J*=12.3 Hz), 6.29 (d, 1H, *J*=9.5 Hz), 6.79-6.86 (m, 1H), 6.89-6.96 (m, 2H), 7.41 (d, 1H, *J*=8.7 Hz), 7.63 (d, 1H, *J*=9.5 Hz), 7.79 (s, 1H), 8.31 (s, 1H); ^{13}C-NMR ($CDCl_3$, 75 MHz) δ 42.4, 44.1, 62.7, 66.8, 84.8, 102.2, 112.5, 113.7, 114.2, 120.0, 129.3, 137.7, 143.1, 150.2, 154.2, 154.7, 155.7, 156.0, 160.4, 160.7; MS (ESI): *m/z* 449 $[M+H]^+$; Anal. Calcd (%) for $C_{22}H_{20}N_6O_5$: C, 58.92; H, 4.50; N, 18.74. Found: C, 59.03; H, 4.46; N, 18.61.

7-{[5-(6-Pyrrolidin-1-yl-9H-purin-9-yl)-4,5-dihydroisoxazol-3-yl]methoxy}-2H-chromen-2-one (18c)

White crystals (60%), m.p. 200-202°C (ethyl acetate); IR (Nujol): 3030, 1705, 1610, 1545 cm^{-1}; ^{1}H-NMR ($CDCl_3$, 300 MHz) δ 1.90-2.04 (m, 4H), 3.67-3.74 (m, 2H), 3.84-4.16 (m, 4H), 5.02 (d, 1H, *J*=12.3 Hz), 5.08 (d, 1H, *J*=12.3 Hz), 6.25 (d, 1H, *J*=9.5 Hz), 6.76-6.84 (m, 1H), 6.84-6.92 (m, 2H), 7.43 (d, 1H, *J*=8.8 Hz), 7.65 (d, 1H, *J*=9.5 Hz), 7.81 (s, 1H), 8.33 (s, 1H); ^{13}C-NMR ($CDCl_3$, 75 MHz) δ 31.2, 42.3, 44.5, 64.4, 84.5, 101.8, 111.5, 112.3, 115.7, 119.8, 127.6, 138.4, 143.6, 150.1, 152.3, 152.6, 154.2, 155.3, 160.6, 161.0; MS (ESI): *m/z* 433 $[M+H]^+$; Anal. Calcd (%) for $C_{22}H_{20}N_6O_4$: C, 61.10; H, 4.66; N, 19.43. Found: C, 61.03; H, 4.59; N, 19.31.

© 2017 Kallitsakis *et al.*

This is an open access article distributed under the terms of the Creative Commons Attribution 4. 0 International Public License (CC-BY 4. 0), a copy of which is available at: https://creativecommons. org/licenses/by/4. 0/legalcode. This license permits unrestricted use, distribution, and reproduction in any medium, provided the original author and source are credited.
